# Ensemble learning-based online sequential pre-interference extreme learning for concept drifting and class imbalanced data streams

**DOI:** 10.1371/journal.pone.0353728

**Published:** 2026-07-31

**Authors:** Yinjie Huang, Hui Wen, Qunhua Tang, Haitao Liu

**Affiliations:** 1 College of Intelligent Manufacturing, Putian University, Putian, Fujian, China; 2 College of Artificial Intelligence, Putian University, Putian, Fujian, China; 3 RunJian Co., Ltd, Nanning, Guangxi, China; Commonwealth Scientific and Industrial Research Organisation, AUSTRALIA

## Abstract

With the rapid development of data-driven technologies, real-time data streams not only exhibit concept drift but are also frequently accompanied by class imbalance problems. To address these challenges, this paper proposes an online sequential pre-interference layer extreme learning machine (OS-PIELM). The proposed model introduces a pre-interference layer between the input layer and hidden layer of the original OS-ELM to enhance nonlinear feature representation through kernel-like transformation of sequential data, thereby improving the discriminative ability of different classes. Furthermore, an adaptive forgetting factor and a Gmean-based concept drift detection mechanism are incorporated into OS-PIELM, together with a dynamic weighting strategy. These components enable the model to effectively handle class imbalance in data streams and enhance its sensitivity to concept drift. Finally, an online ensemble learning framework is constructed with OS-PIELM as the base classifier to further improve the robustness of the proposed method. Extensive experiments on nine synthetic datasets and two real-world datasets demonstrate that the proposed method can effectively address class imbalance in data streams and improve concept drift detection performance.

## Introduction

With the rapid development of the information age, massive data streams are continuously generated in various domains, including fault diagnosis [1–3], satellite remote sensing [4,5], weather forecasting [6,7], and traffic monitoring [8,9]. Unlike traditional static datasets, data streams exhibit high velocity, continuous evolution, and potentially unbounded size. Mining valuable information within data streams has spurred the demand for machine learning techniques, one of which is a classification algorithm. Among these techniques, online learning has emerged as an effective paradigm for handling continuously evolving data streams.

The diverse applications of data streams have driven extensive research in online learning, particularly in scenarios involving incomplete data, evolving environments, and limited labeled samples. To address incomplete or uncertain data, You et al. [10] proposed an online learning algorithm for data streams with incomplete features and labels (OLIFL), which dynamically maintains a global feature space and estimates label confidence for unlabeled instances. This approach effectively improves learning performance under incomplete data conditions. However, it is primarily designed for static data distributions and does not explicitly consider distributional changes or dynamically emerging class structures. To cope with evolving data distributions, Zhuo et al. [11] introduced an adaptive sliding window to detect changes in data streams and maintain model stability. This method enhances the adaptability of models to dynamic environments and improves drift detection capability. Nevertheless, it mainly focuses on distributional variations and does not address the challenges caused by imbalanced class distributions. In addition, Wu et al. [12] explored semi-supervised learning strategies to reduce the reliance on labeled data, thereby improving the applicability of online learning methods. This strategy effectively alleviates the problem of label scarcity and extends the usability of online learning. However, it primarily targets insufficient labeling and does not explicitly consider the interaction between evolving data distributions and skewed class distributions. From a broader perspective, these studies provide important insights into different aspects of data stream learning. Specifically, OLIFL highlights the importance of confidence-aware learning for handling uncertain information, Zhuo et al.’s method demonstrates the effectiveness of adaptive mechanisms in tracking evolving data distributions, and Wu et al. reveals the potential of semi-supervised strategies in alleviating label scarcity. These observations suggest that effectively addressing real-world data stream problems requires the integration of multiple complementary mechanisms.

In particular [13,14], in practical scenarios where data distributions evolve over time and class imbalance is prevalent [15,16], it is necessary to jointly consider distribution adaptation and imbalance-aware learning. Therefore, it is natural to explore a unified learning framework that builds upon these ideas and further models the interaction between concept drift and class imbalance, so as to achieve more robust and reliable performance in dynamic environments.

For classification tasks in data streams with concept drift, a common framework is to detect newly arriving data blocks under either supervised or unsupervised settings, followed by the design of corresponding drift adaptation mechanisms [17]. In contrast to supervised approaches, unsupervised methods typically detect drift by observing distributional differences within data blocks [18]. Comparatively, supervised methods generally achieve higher detection accuracy and efficiency; therefore, this study focuses on supervised drift detection. In most existing frameworks, the adaptation module is triggered only when a new concept is detected [19]. The decision of whether drift has occurred usually relies on a predefined threshold [20], which directly affects the accuracy and efficiency of drift detection. To address this limitation, adaptive forgetting factor mechanisms [21] have been introduced. These methods dynamically adjust the forgetting rate based on the performance variation of the classifier itself, thereby controlling the extent to which outdated concepts are discarded and new data are incorporated into the model. However, most previous studies assume that data streams are class-balanced, which limits their applicability in real-world scenarios where class imbalance is prevalent.

When class imbalance and concept drift coexist, they constitute two distinct yet coupled challenges in data stream classification, rather than a simple superposition of two independent problems. Concept drift may alter class prior probabilities or class-conditional distributions, thereby causing the degree of class imbalance to evolve dynamically over time. In turn, class imbalance further exacerbates the difficulty of learning and classification in drifting data streams, particularly by weakening the model’s ability to recognize minority classes, and may significantly degrade the overall performance of online learning models, including concept drift detection mechanisms. Due to the skewed data distribution, traditional machine learning algorithms often struggle to accurately identify minority class samples that may contain critical information [22]. Recent studies have further demonstrated the limitations of individual machine learning models on small-scale and highly imbalanced real-world datasets. Li et al. [23]reported that conventional machine learning algorithms often show suboptimal predictive performance on imbalanced clinical data collected from local hospitals. Their findings indicate that relying solely on a single classifier may be insufficient under severe class imbalance, highlighting the necessity of developing more robust imbalance-aware and ensemble-based learning approaches. In the absence of prior knowledge about the imbalance ratio, one practical approach is to estimate the imbalance level within each data block and assign higher weights to minority-class instances to mitigate this issue. Mirza et al. proposed the Weighted Online Sequential Extreme Learning Machine (WOS-ELM) [24], which is based on a cost-sensitive learning strategy. In this method, minority-class samples are reweighted according to the imbalance ratio (IR), enabling the classifier to better handle class imbalance. Building upon this work, the authors further developed a voting-based extension of WOS-ELM in 2015 [25], where multiple WOS-ELM classifiers are combined, allowing the approach to be applied to multi-class classification tasks. Motivated by these advances, a natural extension is to integrate concept drift detection, adaptive forgetting mechanisms, and class imbalance-aware weighting strategies into the OS-ELM [26] framework.

OS-ELM is an incremental learning neural network that adopts a block-wise learning strategy, where data are processed in chunks rather than individually. In online learning scenarios, the temporal variability and weak correlation of incoming samples make it difficult for the algorithm to effectively capture the underlying spatial distribution of the data. Within drift detection modules, error-rate-based methods are commonly used as indicators to determine whether concept drift has occurred. However, in the presence of class imbalance, where majority-class samples dominate, this approach becomes less reliable. Even if the model completely misclassifies minority-class instances, the overall error rate may remain low or exhibit only minor fluctuations as long as the majority-class predictions are correct. Moreover, existing methods typically design concept drift detection, class imbalance handling, and model updating as separate modules, without sufficient interaction among them. This decoupled design overlooks the intrinsic relationship between concept drift and class imbalance, thereby limiting the adaptability and overall performance of the model in complex data stream environments. To better illustrate the research gaps, the main challenges in class-imbalanced data stream learning with concept drift are summarized as follows:

- **Insufficient coupling among functional modules**: Existing methods often treat concept drift detection, class imbalance handling, and model updating as independent components, leading to weak interaction among modules and suboptimal overall performance.
- **Unreliable drift detection under class imbalance**: Error-rate-based drift detection methods may become ineffective in imbalanced scenarios, as the misclassification of minority-class samples can be masked by the dominance of majority-class instances.
- **Difficulty in modeling dynamic and unstable data distributions**: The temporal variability and weak correlation of streaming data make it challenging for models to capture stable patterns, thereby reducing robustness and generalization ability.

To address these challenges, we propose a new online learning algorithm for data streams that simultaneously handles class imbalance and concept drift. First, we enhance the standard OS-PIELM classifier by inserting a pre-interference layer between its input and hidden layers. This layer applies a nonlinear transformation to each incoming data block, mitigating nonstationary behavior and weak inter-block correlations. Next, to combat class imbalance, we compute the ratio of majority to minority samples within each block and assign adaptive weights to minority instances, ensuring the classifier remains sensitive to underrepresented classes without prior knowledge of the imbalance ratio. We then use the classifier’s Gmean on each block as a drift indicator. When performance drops significantly, the model automatically shifts its focus between current and historical blocks to accommodate distributional changes. Finally, we wrap OS-PIELM in an online ensemble framework that combines multiple models via cooperative voting and incremental updates, boosting both classification accuracy and generalization on continuously arriving data.

1. A novel online learning classifier: OS-PIELM, is created by inserting a pre-interference layer between the input and hidden layers of the standard OS-ELM. This layer applies a nonlinear kernel mapping to sequential online samples, reducing raw-data complexity, enhancing feature separability, and easing the burden of output-weight optimization, thereby boosting data-stream learning performance.
2. To mitigate the impact of class imbalance on concept-drift detection, we propose a Gmean-based concept drift detector coupled with an adaptive forgetting factor mechanism. The forgetting factor is adjusted adaptively according to the ratio between the model’s current Gmean and its historical maximum Gmean.
3. We integrate the adaptive forgetting factor and a weighting strategy into OS-PIELM to enable simultaneous handling of concept drift and class imbalance in data streams.
4. Using OS-PIELM as the classifier, we propose an OS-PIELM–based ensemble algorithm. By employing weighted voting and component updates, the ensemble enhances inter-component coupling and improves the algorithm’s stability.

The rest of this paper is organized as follows. Section 2 reviews the OS-ELM algorithm and highlights key advances in concept drift detection and handling imbalanced data. Section 3 details our OS-PIELM classifier, describing its Gmean-based drift detection mechanism with an adaptive forgetting factor, the dynamic weighting strategy, and the online ensemble algorithm. In Section 4, we compare our method with state-of-the-art online learners on both synthetic and real-time datasets that exhibit concept drift and class imbalance and analyze the results. Finally, Section 5 summarizes our main contributions and suggests directions for future work.

## Related work

This section begins by explaining the principles of OS-ELM and then provides a comprehensive review of concept drift detection methods and related research on handling data imbalance.

### OS-ELM

The OS-ELM algorithm was introduced by Liang et al. Its training process consists of two stages: an initialization phase and an online sequential learning phase. First, a small batch of data is used to compute the initial output matrix *H*_0_ and output weights $\beta_{0}$; thereafter, these parameters are iteratively updated as new input samples arrive. The detailed procedure is as follows:

Given a dataset $S\;=\;\{\;(x_{i},y_{i},)\;|\;x_{i}\in R^{n},\;y_{i}\in R^{m},\;i=1,2,\ldots ,N\}.$ where each $x_{i}$ is an n*1 input vector (with *n* denoting its dimensionality) $y_{i}$ is an m*1 target vector (where *m* is the number of classes), and *N* is the total number of samples. We partition *S* into *k* disjoint data blocks; denote the k-th block by $x_{i},y_{i}$ and let $N_{k}$ be the number of samples in that block. Select the first *N*_0_ samples, $(x_{i},\;y_{i})\;{\}}_{i=1}^{N_{0}}$ as the initial block, where *N*_0_ is the number of initial training samples. In the initialization phase, randomly generate the input-to-hidden weights and biases $(x_{i},\;y_{i})\;{\}}_{j=1}^{L}$ where *L* is the number of hidden‐layer nodes and *j* indexes the j−th hidden node. Using the initial samples $(x_{i},\;y_{i})\;{\}}_{i=1}^{N_{0}}$, construct the initial hidden‐layer output matrix


$$\begin{array}{c} H_{0}\;=\;{\left(\begin{array}{ccc} G(\omega_{1},\;b_{1},\;x_{1}) & \cdots & G(\omega_{L},\;b_{L},\;x_{1})\\ \vdots & \ddots & \vdots \\ G(\omega_{1},\;b_{1},\;x_{N_{0}}) & \cdots & G(\omega_{L},\;b_{L},\;x_{N_{0}}) \end{array}\right)}_{R^{N_{0}\times L}}\; \end{array}$$ (1)

where $G(\omega_{j},\;b_{j},\;x_{i})$ is the activation output of the *j*-th hidden node given input $x_{i}$. According to [27], the initial output weight $\beta_{0}$ is defined as:


$$\begin{array}{c} \beta_{0}\;=\;P_{0}\;H_{0}^{T}\;T_{0}, \end{array}$$ (2)

where *P*_0_ denotes the initial auxiliary matrix, in the initialization phase, it is defined as $P_{0}\;=\;(H_{0}^{T}H_{0}{)}^{-1},T_{0}\;=\;{\left[\begin{array}{c} t_{1},\;\ldots ,\;t_{N_{0}} \end{array}\right]}^{T}.$

During the online sequential learning phase, OS-ELM processes data in blocks. Suppose that when the k-th data block arrives, the hidden-layer output matrix $H_{k}$ and the corresponding target matrix $T_{k}$ are redefined as:


$$\begin{array}{c} H_{k}\;=\;G(\omega_{1},\;\ldots ,\;\omega_{L},\;b_{1},\;\ldots ,\;b_{L},\;x_{1},\;\ldots ,\;x_{\sum_{i=1}^{k}N_{i}}), \end{array}$$ (3)

$$\begin{array}{c} T_{k}\;=\;{\left[\begin{array}{c} t_{1},\;\ldots ,\;t_{\sum_{i=1}^{k}N_{i}} \end{array}\right]}^{T}, \end{array}$$ (4)

where $N_{i}$ is the number of samples in the *i*-th block. When the (*k* + 1)-th block arrives, these definitions become:


$$\begin{array}{c} H_{\;k+1}\;=\;G(\omega_{1},\;\ldots ,\;\omega_{L},\;b_{1},\;\ldots ,\;b_{L},\;x_{1},\;\ldots ,\;x_{\sum_{i=1}^{k}N_{i}},\;\ldots ,\;x_{\sum_{i=1}^{\;k+1}N_{i}}), \end{array}$$ (5)

$$\begin{array}{c} T_{\;k+1}\;=\;{\left[\begin{array}{c} t_{1},\;\ldots ,\;t_{\sum_{i=1}^{k}N_{i}},\;\ldots ,\;t_{\sum_{i=1}^{\;k+1}N_{i}} \end{array}\right]}^{T}. \end{array}$$ (6)

At this point, the auxiliary matrix $P_{k}$ and output weight vector $\beta_{k}$ are updated incrementally as new data chunks arrive. The recurrence relations are expressed as:


$$\begin{array}{c} P_{k+1}=P_{k}\;-\;P_{k}\;H_{k+1}^{T}(I+H_{k+1}\;P_{k}\;H_{k+1}^{T}{)}^{-1}H_{k+1}\;P_{k} \end{array}$$ (7)

$$\begin{array}{c} \beta_{k+1}=\beta_{k}\;+\;P_{k+1}\;H_{k+1}^{T}(T_{t+1}^{T}-H_{k+1}\;\beta_{k}) \end{array}$$ (8)

OS-ELM combines the speed and generalization advantages of ELM. As shown in Eqs. (7) and (8), its output weights are iteratively updated using the most recent result and incoming data, allowing the model to evolve continuously without retraining. This update strategy significantly reduces computational overhead and memory usage, making it ideal for online learning scenarios. Consequently, optimizing algorithms around OS-ELM has become a popular research direction in data stream classification.

### Drift detection mechanism

**Fig 1:**
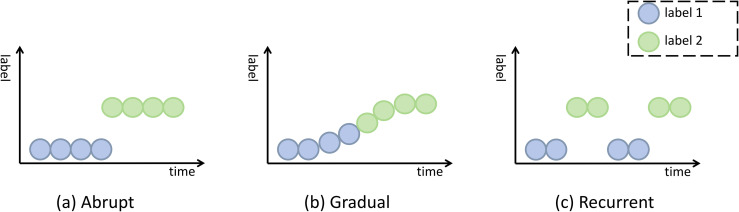
Concept drift types.

In data streams, instances typically arrive at high speed and in unbounded volume. However, their underlying distribution or the associated labels may evolve, represented by $P_{t}(X,y)\text{≠}P_{t+1}(X,y)$ [28], it signifies the emergence of a new concept. Here, *X* denotes a two-dimensional feature space, and *y* represents its corresponding label [29]. The Fig 1 illustrates three types of concept drift.

Research on concept drift is extensive and can be broadly divided into three categories: error rate, data distribution and hypothesis testing. DDM (Drift Detection Method) [30,31] and ECDD (Exponentially Weighted Moving Average Concept Drift Detection) [32] are typical error rate based algorithms that continuously feed all incoming training samples into the model and have demonstrated strong classification performance. Specifically, within a sliding time window, they compute the error rate between each new sample’s accurate label and its predicted label—an indicator known as the ’online average error rate’. When this error rate exceeds a predefined warning threshold, the system triggers and constructs a new classifier; only if the error rate further rises to reach the ’drift’ threshold does the new classifier replace the existing model. This mechanism ensures that the current model remains in use until an actual concept drift occurs.

Different from the above methods, conceptual drift detection based on data distribution treats the data of the past period (the reference window) and the latest arriving period of data (the detection window) as two sets of samples and determine whether their distributions have undergone a significant difference using a statistical test or a distance metric: if the two sets of data in terms of characteristic distributions (e.g., probability distributions of dimensions) or output distributions (e.g., class frequencies) the difference exceeds a predetermined threshold, concept drift is considered to have occurred. Otherwise, it is regarded as normal sampling fluctuation. For example, Guo et al [33] proposed a local drift detection for clustering, which compares the differences in data distributions between historical and current data blocks, and is used to capture new concepts and identify the corresponding drifted regions. Li et al [34] used Gaussian Mixture Models (GMMs) to obtain similarities between data distributions, which are used to assign weights to the base learner for fast adaptation to conceptual drifts.

Hypothesis testing methods employ statistical tests to determine whether observed errors (or distribution changes) are merely random fluctuations due to sampling variability or indicate genuine concept drift. For example, Sun et al [35] proposed two such detection techniques: EBTBM (Entropy-Based Thresholding Method) and EBBSM (Entropy-Based Sampling Method). Both approaches employ the uncertainty inherent in the target concept’s definition as a statistical measure of the difference between two consecutive data blocks.

### Class imbalanced learning

In data streams, a common issue is class imbalance within the dataset, where one or more classes are significantly underrepresented compared to others. This imbalance becomes particularly problematic when the minority class carries more critical information since models built on such data incur higher classification costs in real-time applications [36]. Furthermore, imbalance not only complicates the construction of effective classifiers but also necessitates specialized algorithms to mitigate bias and ensure robust performance in all classes. Consequently, designing classifiers for imbalanced datasets remains a significant challenge in data mining.

To the best of our knowledge, popular approaches to addressing class imbalance can be categorized into three types: data-level, algorithm-level, and hybrid strategies [37]. At the data level, random undersampling and oversampling are two commonly used techniques. They balance class proportions by either reducing the number of majority‐class samples or increasing the number of minority‐class samples. Payel Sadhukhan [38] introduced a novel undersampling strategy applied to synthetic minority sets. Sun et al. [39] proposed an undersampling method that incorporates minority‐class density information: they use kernel density estimation to learn the probability density of minority samples, filter out majority samples located in high‐density regions of the minority class, and then define a ’sampling fitness’ measure to evaluate each majority sample’s desirability—thereby selecting the most informative examples. Others [40] have proposed an adaptive weighting and nearest‐neighbor–based region‐control method to address insufficient noise filtering in oversampling, difficulty in interpreting sample importance, and exacerbated class overlap.

Unlike the data-level approaches, algorithm-level methods start by modifying the classification algorithm itself. Zhu et al. [41] addressed the imbalanced data problem by considering class distribution through a novel hybrid resampling method, MSHR, and an improved cost-sensitive SVM model. Similarly, Liang et al. [42] introduced a pre-grouping strategy into the SVM framework to optimize data distribution and enhance the quality of minority-class samples. Moreover, Dai et al. [43] proposed a genetic algorithm that selects the optimal combination of heterogeneous clustering techniques—guided by fitness functions—to resolve class-overlap problems.

Hybrid strategies combine the advantages of both data-level and algorithm-level methods. Li et al. [44] incorporated the density information of training samples into the class‐imbalance ratio, thereby assigning different weights to samples even within the same class. By adopting the Learn++ algorithm [45], Ditzler et al. [46] further adjusted each base classifier’s voting weight according to the imbalance ratios observed in both current and historical data.

More and more research focuses on imbalanced learning [47], especially in real-world applications. Mingkuan Shi et al. [48] addressed imbalanced industrial data streams by leveraging prior distribution information to strengthen the classification decision boundary between majority and minority classes, thereby proposing a novel imbalance-aware learning system. Similarly, Wu et al [49] employed a feature‐based resampling approach to correct classifier bias induced by class imbalance.

**Algorithm 1 The flowchart of EOS‐PIELM**

**Input:**

*S*: data stream

$\{x_{i},\;y_{i}{\}}_{i=1}^{N_{k}}$: *k*‐th data block

*E*: ensemble classifier

*M*: number of base classifiers

**Output:**

new(E): updated *E*

1: perform $\{x_{i},\;y_{i}{\}}_{i=1}^{N_{0}}\;\to \;\text{dynamic weighting strategy}$

2:  $\{x_{i},\;t_{i}{\}}_{i=1}^{N_{0}}$ by $E\to P_{0}$

3:  **while** S→∞ **do**

4:   perform $\{x_{i},\;y_{i}{\}}_{i=1}^{N_{k}}\;\to \;\text{dynamic weighting strategy}$

5:   $\{x_{i},\;y_{i}{\}}_{i=1}^{N_{k}}$ by $E\to P_{1}$

6:   **if** perform drift detection → *P*_0_ compared *P*_1_ **then**

7:    perform drift adjust → update λ

8:    perform adjust CF

9:   **end if**

10:   **return** λ and CF → update new(E)

11:  **end while**

## Proposed method

**Fig 2:**
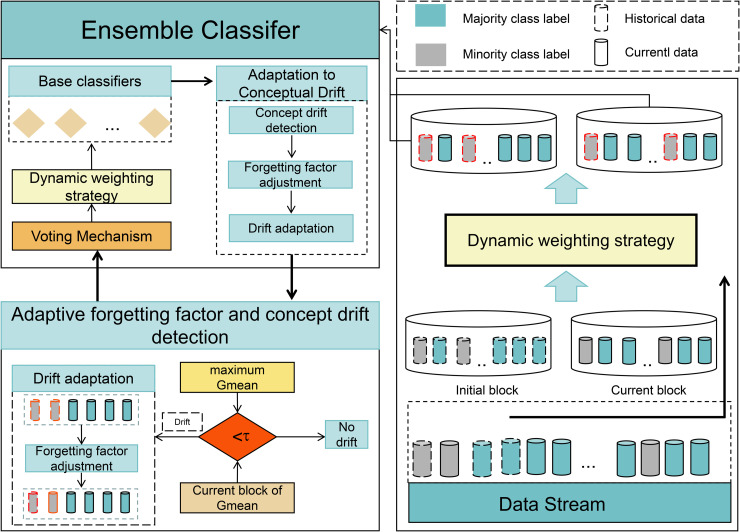
The flowchart of EOS-PIELM.

This section presents a novel framework for addressing concept drift and class imbalance in dynamic data streams, as illustrated in Fig 2. First, *N*_0_ samples are drawn from the beginning of the historical data stream to form the initial training set for the ensemble classifier. The ensemble model, denoted as $E=\{\;f_{1},f_{2},\ldots ,f_{M}\}$, consists of M heterogeneous OS-PIELM base classifiers. Then, a voting mechanism is used to select the classification result with the highest score. Whenever new samples accumulate to a predetermined threshold, they are grouped into a new data block, and the current ensemble model is used to predict its labels by vote. Next, a based Gmean drift detector is triggered: the maximum Gmean value among all previous data blocks is compared with the Gmean of the new block, and if their difference exceeds a preset threshold, concept drift is declared. The magnitude of this drift determines the forgetting factor, which in turn controls the weight assigned to past samples. Based on both the forgetting factor and each base learner’s recent performance, voting weights are adjusted for all classifiers. To address class imbalance, the class distribution of each data block is assessed before prediction, and minority-class samples are assigned a greater weight. After each round, a dynamic cost factor (CF) is introduced for every base classifier: CF adjusts that classifier’s focus on minority-class instances according to its performance and the current forgetting factor, and the network parameters are updated via weighted recursive least squares to maintain the model’s online learning capability and preserve discrimination power for minority classes. Detailed procedural steps are provided in Algorithm 1.

### OS-PIELM

To improve the nonlinear representation capability of ELM, various feature mapping methods have been proposed, among which Kernel ELM [50] and random feature-based ELM [51] are representative approaches. By mapping the original samples into a high-dimensional feature space, these methods can effectively enhance class separability and improve the approximation ability of ELM for complex nonlinear problems. Kernel ELM enhances nonlinear representation by constructing a kernel matrix that characterizes pairwise relationships among training samples. Consequently, the feature representation depends on the entire training dataset, and the nonlinear mapping process is achieved through kernel matrix operations. Although this strategy can improve classification performance, it requires kernel matrix construction and storage, making it difficult to efficiently accommodate continuously arriving data in online sequential learning scenarios. Random feature-based ELM methods improve nonlinear representation by projecting original samples into a higher-dimensional space through randomly generated mapping parameters. While these methods avoid explicit kernel matrix construction, their mapping performance is strongly influenced by randomly initialized parameters and generally follows a fixed transformation mechanism after model initialization.

**Fig 3:**
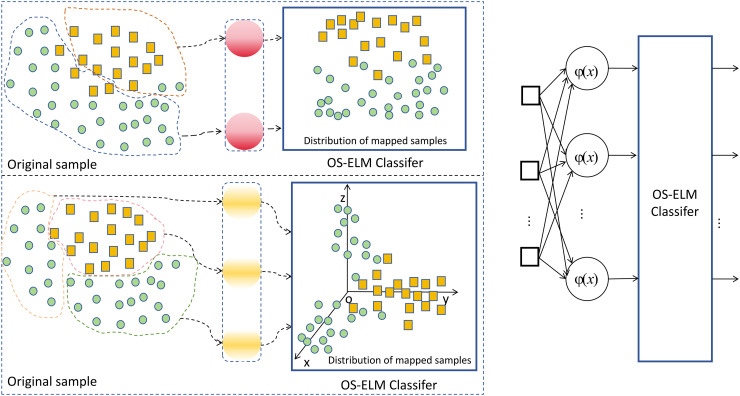
Kernel mapping process.

In addition, several online feature-mapping methods, such as Online Sequential Kernel ELM (OS-KELM) [52] and online random feature-based learners, extend nonlinear feature mapping to streaming environments. However, these methods still rely on either kernel-based sample relationship modeling or randomly generated feature transformations. Different from the above approaches, the proposed pre-interference layer neither constructs a kernel matrix nor employs random feature mapping. Instead, it directly performs nonlinear feature reconstruction on each incoming sample before hidden-layer learning. Specifically, a set of interference nodes is introduced to transform the original input sample into a new feature representation, thereby enhancing feature separability in the transformed space. Since the transformation is performed on individual samples rather than on pairwise sample relationships, the proposed method does not depend on the entire training dataset and can naturally preserve the sequential updating mechanism of OS-ELM. Motivated by this idea, a novel pre-interference layer is inserted between the input layer and hidden layer of the original OS-ELM network, resulting in a four-layer feedforward neural network architecture. The layer consists of a set of sigmoid kernel functions, where all connection weights between the input layer and the enhancement layer are set to 1. when an input sample *x* passes through this interference layer, it is transformed into φ(x) into a new representation (see Fig 3). In the figure, the upper part contains a pre-interference layer with two nodes, whereas the lower part contains a pre-interference layer with three nodes. The orange rectangles and green circles represent two different classes of sample data points. It can be observed that, through nonlinear kernel mapping of the original samples, the distribution complexity of the samples in the kernel space is improved. From the perspective of function approximation, a single hidden layer network theoretically possesses universal approximation capability. However, under the condition of a limited number of hidden nodes, the approximation efficiency depends on the complexity of the target function in the input space. If the mapping can nonlinearly reconstruct the original samples so that they exhibit a clearer class structure in the new space, then the complexity of the target function in the mapped space is reduced. This helps alleviate the optimization burden of the output weights in the subsequent OS-ELM network.

From the perspective of feature separability, let the inter-class distance between two classes in the original space be $\left\|\mu_{1}-\mu_{2}\right\|$, and the intra-class scatter be $S_{1}+S_{2}$. The discriminative ability can be expressed using the Fisher discriminant ratio as:


$$\begin{array}{c} J=\frac{\|\mu_{1}-\mu_{2}{\|}^{2}}{S_{1}+S_{2}} \end{array}$$ (9)

The subspace mapping is a kind of feature space transformation. If the mapping increases the inter-class distance and reduces the intra-class variance, then the discriminant ratio *J* in the transformed space increases, thereby improving the separability of the samples. In this case, OS-ELM performs linear combination in this space, which enables more stable classification and regression.

**Algorithm 2 OS‐PIELM**

**Input:**

$\{x_{i},y_{i}{\}}_{i=1}^{N_{0}}$: initial data block

$\{x_{i},y_{i}{\}}_{i=1}^{N_{k+1}}$: *k* + 1-th data block

φ(·): pre-interference layer activation function

G(·): hidden layer activation function

**Output:**

$new(\beta_{k+1}):\beta_{0}\to \beta_{k+1}$

**Initialization:**

1:  perform pre-interference $\left\{x_{i},y_{i}{\}}_{i=1}^{N_{0}}\;\to \;\varphi \;(\sum_{i=1}^{N_{k+1}}x_{i}\right)$

2:  by eq. (9): $\varphi \;(\sum_{i=1}^{N_{k+1}}x_{i})\to H_{0}$

3:  by eq. (2): $H_{0}\to \beta_{0}$

**Online learning:**

1:  **while** $N_{0}\to N_{k+1}$ **do**

2:   perform pre-interference $\left\{x_{i},y_{i}{\}}_{i=1}^{N_{0}}\;\to \;\varphi \;(\sum_{i=1}^{N_{k+1}}x_{i}\right)$

3:   by eq. (10): $\varphi \;(\sum_{i=1}^{N_{k+1}}x_{i})\to H_{k+1}$

4:   **if** $N_{k+1}=1$ **then**

5:    by eqs. (11) and (12): $H_{k+1}\to \beta_{k+1}$

6:   **else**

7:    by eqs. (7) and (8): $H_{k+1}\to \beta_{k+1}$

8:   **end if**

9:   **return** $new(\beta_{k+1})$

10:  **end while**

By using φ(x) as the input vector to the OS-PIELM hidden layer, m samples are chosen during the initialization phase to recompute the initial output matrix, which is expressed as:


$$\begin{array}{c} H_{0}\;=\;{\left(\begin{array}{ccc} G(\omega_{1},\;b_{1},\;\varphi (x_{1})) & \cdots & G(\omega_{L},\;b_{L},\;\varphi (x_{1}))\\ \vdots & \ddots & \vdots \\ G(\omega_{1},\;b_{1},\;\varphi (x_{N})) & \cdots & G(\omega_{L},\;b_{L},\;\varphi (x_{N})) \end{array}\right)}_{\;N\times L}. \end{array}$$ (10)

When the hidden‐layer output matrix *H*_0_ is substituted into equation (2), the initial output weight $\beta_{0}$ can be obtained.

In the context of online sequential learning—where data arrive in batches and exhibit time‐varying characteristics with weak inter‐sample correlations—each time a new (k + 1)-th data batch becomes available, we construct the hidden‐layer output matrix for that batch as follows:


$$\begin{array}{c} H_{k+1}\;=\;{\left(\begin{array}{ccc} G(\omega_{1},\;b_{1},\;\varphi (x_{1})) & \cdots & G(\omega_{L},\;b_{L},\;\varphi (x_{1}))\\ \vdots & \ddots & \vdots \\ G(\omega_{1},\;b_{1},\;\varphi (x_{N_{k+1}})) & \cdots & G(\omega_{L},\;b_{L},\;\varphi (x_{N_{k+1}})) \end{array}\right)}_{\;N_{k+1}\times L}. \end{array}$$ (11)

At this point, we update the inverse‐correlation matrix $P_{k+1}$ and the output weight $\beta_{k+1}$ using the recursive formulas Eqs. (7) and (8). In practice, the size of the (k + 1)-th batch $\left(N_{k+1}\right)$ may differ from one. When $N_{k+1}$ following references [53,54], equations Eqs. (7) and (8) can be rewritten in scalar‐update form as


$$\begin{array}{c} \beta_{\;k+1}\;=\;\beta_{\;k}\;+\;P_{\;k}\;h_{\;k+1}\;(t_{\;k+1}^{T}\;-\;h_{\;k+1}^{T}\;\beta_{\;k}), \end{array}$$ (12)

$$\begin{array}{c} P_{\;k+1}\;=\;P_{\;k}\;-\;\frac{P_{\;k}\;h_{\;k+1}\;h_{\;k+1}^{T}\;P_{\;k}}{\;1\;+\;h_{\;k+1}^{T}\;P_{\;k}\;h_{\;k+1}\;}\;. \end{array}$$ (13)

where the $h_{k+1}\;=\;(\;G(\omega_{1},\;b_{1},\;\varphi (x_{k+1}))\;,\;\ldots ,\;G(\omega_{L},\;b_{L},\;\varphi (x_{k+1}))\;).$ After all incoming data batches have been processed, the final output weight $\beta_{k+1}$ is obtained, yielding the complete OS-PIELM model. Detailed procedural steps are provided in Algorithm 2.

Although the pre-interference layer in OS-PIELM can be used to perform nonlinear kernel mapping on the online sequential samples to enhance the discrimination of different features, the class labels of the data may change rapidly after the data stream undergoes conceptual drift, and the original model cannot respond and update in a timely manner; At the same time, if the original data suffers from severe class imbalance, the majority-class samples mapped into the high-dimensional space will still dominate, which biases the classification boundary toward the majority class and weakens the model’s ability to recognize the minority class, the majority class samples mapped into the high-dimensional space by the pre-interference layer will still dominate, which further biases the classification boundary towards the majority class and weakens the model’s ability to recognize the minority class. In order to ensure that the model can quickly respond to the changes of distribution and labeling in the data stream while taking into account the detection performance of the minority class samples, it is necessary to introduce a concept drift detection and class imbalance learning strategy.

### Adaptive forgetting factor and concept drift detection

When data drift occurs, sample labels tend to change. Overstoring historical information only reduces the model’s ability to predict new data. To address this, we introduce a forgetting factor λ in the hidden-layer-output weight β update of OS-PIELM and, on that basis, derive a new update formula. This ensures that the algorithm can adapt quickly and maintain classification performance when facing concept drift.

By incorporating the forgetting factor into Eq. (10), so that new and old data batches carry different weights, Eq. (10) becomes:


$$\begin{array}{c} P_{k+1}\;=\;\frac{1}{\lambda }P_{k}\;-\;\frac{1}{\lambda^{2}}\;P_{k}\;H_{k+1}^{T}(I+\frac{1}{\lambda }\;H_{k+1}P_{k}H_{k+1}^{T}{)}^{-1}H_{k+1}\;P_{k}. \end{array}$$ (14)

When λ=1, the model degenerates to the original OS-PIELM.

Although in theory OS-PIELM can accommodate various degrees of concept drift by adjusting the forgetting factor, it still suffers from several shortcomings:

1. A fixed forgetting factor cannot strike a balance between discarding outdated information and retaining useful historical knowledge.
2. While it adapts well to gradual drift (slow, continuous changes in the data distribution), it cannot adjust quickly enough during abrupt drift (especially concept reversals), resulting in delayed updates and a significant drop in classification performance.
3. In most real-time datasets, the type of concept drift is unknown, so it is impossible to tune the forgetting factor optimally for each specific drift pattern.
4. Compared with classical drift detection methods such as DDM and ADWIN, the proposed Gmean-based mechanism exhibits distinct advantages in imbalanced data stream scenarios. DDM primarily relies on monitoring the overall error rate, which may be dominated by the majority class and thus fail to reflect performance degradation on minority classes. ADWIN detects changes by analyzing data distribution shifts within adaptive windows, but it does not explicitly consider class-wise performance.

**Algorithm 3 Based Gmean Drift Detection Mechanism**

**Input:**

λ: default forgetting factor

τ: concept drift detection threshold

${Gmean}_{k}$: predicted Gmean for the *k*-th data block

${Gmean}_{\max}$: historical max Gmean

*i*: total number of data blocks

**Output:**

new(λ): updated λ

1:  **for** *k* = 1 **to** *i* **do**

2:   Compare ${Gmean}_{k}$ and ${Gmean}_{\max}$

3:   **if** $|{Gmean}_{k}-{Gmean}_{\max}|>\tau$ **then**

4:    $CDI=\frac{{Gmean}_{k}}{{Gmean}_{\max}}$

5:    **if** CDI≤0.9 **then**

6:     λ=0.9+0.1×CDI

7:    **else**

8:     λ=0.999

9:    **end if**

10:   **end if**

11:  **end for**

To address the above issues, we incorporate a concept drift detection mechanism based on the geometric mean (Gmean) into OS-PIELM. Since Gmean simultaneously captures the classification performance of both positive and negative classes and reflects the balance between majority and minority classes, it is less sensitive to performance fluctuations dominated by the majority class compared to metrics that rely solely on overall error rate. As a result, it can effectively suppress spurious drift alarms caused by transient variations in error rates, while also mitigating false alarms and missed detections commonly observed in imbalanced data scenarios. When the Gmean value falls below a predefined threshold, concept drift is detected, and the decay rate of the forgetting factor is adaptively increased. This mechanism assigns higher weights to newly arriving samples, thereby enabling the model to respond more rapidly to abrupt environmental changes. The derivation for setting this threshold is as follows:

By applying Hoeffding’s inequality to concept drift detection, the relationship between the allowable performance degradation threshold and the sample size *n* at significance level δ is derived:


$$\begin{array}{c} P\left(\left|\bar{X}-E[\bar{X}]\right|>\epsilon \right)\leq \delta , \end{array}$$ (15)

where $\delta =2\exp(-2n\epsilon^{2})$, setting the right-hand side equal to the significance level ϵ yields:


$$\begin{array}{c} \epsilon =\sqrt{\frac{\ln(2/\delta )}{2n}}, \end{array}$$ (16)

this ϵ controls the detection confidence level. Considering the historical maximum Gmean_max as the expected performance μ, and define the relative deviation to characterize the magnitude of performance degradation:


$$\begin{array}{c} \epsilon_{r}=\frac{\epsilon }{\mu }. \end{array}$$ (17)

Using typical parameters δ=0.05 and n∈[50,200], the threshold τ is obtained as:


$$\begin{array}{c} \tau =1-\epsilon_{r}\approx 0.9. \end{array}$$ (18)

In the OS-PIELM algorithm, the default forgetting factor is initialized at λ=0.999. When the Concept Drift Index (CDI) satisfies CDI ≤0.9, the forgetting factor is updated according to:


$$\begin{array}{c} \lambda =0.9+\text{CDI}\times 0.1, \end{array}$$ (19)

where the Concept Drift Index is defined as $\text{CDI}=\text{Gmean }/{\text{Gmean}}_{\max}$. Consequently, under concept drift detection, the λ ranges within [0.9, 0.99], with the update rule defined as:


$$\begin{array}{c} \lambda =\left\{ \begin{array}{cc} 0.9+\text{CDI}\times 0.1, & \text{CDI}\leq 0.9\;\text{(Drift)}\\ 0.999, & \text{CDI}>0.9\;\text{(Stability)} \end{array}\right.. \end{array}$$ (20)

When concept drift leads to a decline in classifier performance, the CDI value decreases, thereby reducing the forgetting factor. Unlike traditional drift detection methods that mainly rely on changes in classification accuracy or error rate, the proposed approach uses Gmean as the drift evaluation metric. Since Gmean considers the recognition performance of both majority and minority classes, the drift detection process can not only reflect distribution changes caused by concept drift, but also capture performance degradation induced by class imbalance. Therefore, the proposed Gmean-based drift detector can uniformly evaluate the model state in imbalanced data stream environments and provide more effective feedback for subsequent adaptive model adjustment. The detected drift information is further used to adaptively adjust the forgetting factor, enabling the model to more appropriately balance the influence of historical data and newly arrived data. steps are provided in Algorithm 3.

### Dynamic weighting strategy

In real‐world applications, class imbalance and concept drift often occur simultaneously. Concept drift causes the data distribution and class labels to change over time. Under class imbalance—where the number of majority‐class samples far exceeds that of the minority class—traditional classifiers tend to shift the decision boundary toward the majority class, reducing sensitivity to the minority class. In this scenario, misclassifying minority‐class instances is not only easier to overlook but also typically incurs a higher cost than misclassifying majority‐class instances. To address this, we build on concept drift detection by incorporating a dynamic weighting strategy: by assigning weights to both the initial training samples and incoming online samples, we mitigate the bias introduced by class imbalance.

During the initialization phase, the imbalance ratio (IR) is computed from the proportion of minority class samples to majority class samples in the initial data chunk. The minority class sample weight *W*_0_ is set to IR, while the majority class weight is fixed at 1. This weighting scheme amplifies the influence of minority class samples on model fitting within the initial matrix *H*_0_. Using the recursive least squares (RLS) method, we derive the recurrence relation for $\beta_{0}$:


$$\begin{array}{c} \beta_{0}={\left(H_{0}^{T}W_{0}H_{0}+CI\right)}^{-1}H_{0}^{T}W_{0}Y_{0} \end{array}$$ (21)

Where $W_{k}=\mathrm{diag}([w_{1},w_{2},\cdots ,w_{k}])$ is the diagonal weight matrix, *C* denotes the regularization parameter, *I* represents the identity matrix, and $Y_{k}=[y_{1},y_{2},\cdots ,y_{k}{]}^{T}$ is the target vector.The initial output weight $\beta_{0}$ is therefore given by:


$$\begin{array}{c} P_{0}={\left(H_{0}^{T}W_{0}H_{0}+CI\right)}^{-1} \end{array}$$ (22)

$$\begin{array}{c} \beta_{0}=P_{0}H_{0}^{T}W_{0}Y_{0} \end{array}$$ (23)

During the online learning phase, when a new batch of examples $\{(x_{i},y_{i}){\}}_{i=1}^{N_{k+1}}$ arrives, we first calculate the class imbalance ratio ${IR}_{k+1}$ for the current data block and assign the sample weight $w_{k+1}$ as:


$$\begin{array}{c} w_{k+1}\;=\;\left\{ \begin{array}{cc} {IR}_{k+1}, & y_{k+1}=0,\\ 1, & y_{k+1}=1. \end{array}\right. \end{array}$$ (24)

Then we construct the hidden-layer output vector:


$$\begin{array}{c} H_{k+1}=\left[G(\omega_{1},b_{1},\varphi (x_{k+1})),\;G(\omega_{2},b_{2},\varphi (x_{k+1})),\;\cdots ,\;G(\omega_{L},b_{L},\varphi (x_{k+1}))\right], \end{array}$$ (25)

using the Woodbury matrix identity, the recursive update formula for the projection matrix $P_{k+1}$ is derived:


$$\begin{array}{cc} P_{k+1} & =(\lambda \;H_{k+1}^{T}\;W_{k+1}\;H_{k+1}\;+\;\lambda \;C\;I{)}^{-1}\\ & =(\lambda \;H_{k}^{T}\;W_{k}\;H_{k}\;+\;{w_{k+1}}_{k+1}^{T}\;H_{k+1}\;+\;\lambda \;C\;I{)}^{-1}\\ & =\frac{1}{\lambda }\;P_{k}\;-\;\frac{w_{k+1}}{\lambda^{2}}\;P_{k}\;h_{k+1}^{T}(\;I\;+\;\frac{w_{k+1}}{\lambda }\;H_{k+1}\;P_{k}\;H_{k+1}^{T}{)}^{-1}\;h_{k+1}\;P_{k}, \end{array}$$ (26)

the recursive update for the output weight $\beta_{k+1}$ during online learning is:


$$\begin{array}{cc} \beta_{k+1} & =(\lambda \;H_{k+1}^{T}\;W_{k+1}\;H_{k+1}\;+\;\lambda \;C\;I{)}^{-1}\;(\lambda \;H_{k+1}^{T}\;W_{k+1}\;Y_{k+1})\\ & =P_{k+1}\;(\lambda \;H_{k}^{T}\;W_{k}\;Y_{k}\;+\;w_{k+1}\;H_{k+1}^{T}\;y_{k+1})\\ & =\beta_{k}\;+\;P_{k+1}\;w_{k+1}\;H_{k+1}^{T}\;(y_{k+1}\;-\;H_{k+1}\;\beta_{k}), \end{array}$$ (27)

where, $W_{k+1}=[\begin{array}{cc} W_{k} & 0\\ 0 & w_{k+1} \end{array}],$ $Y_{k+1}=[\begin{array}{cc} Y_{k} & 0\\ 0 & y_{k+1} \end{array}].$

### Online ensemble learning algorithm

To enhance algorithm robustness for complex real-time data streams, we propose an online ensemble EOS-PIELM algorithm. Building upon the OS-PIELM base classifier, this approach integrates an adaptive forgetting factor, concept drift detection mechanism, and dynamic weighting strategy.

At initialization phase, we construct an ensemble of M OS-PIELM base classifiers. Each base classifier has a different number of pre-interference layer neurons and hidden neurons, specifically [inputs,5×inputs,10×inputs,…,m×inputs], and each uses one of the activation functions sigmoid,softplus,tanh, in this experiment, M was set to 12. By combining different hidden‐layer sizes with different activation functions, we ensure that the base classifiers differ both in structural complexity and in their nonlinear‐mapping capabilities.

Given a data stream of *N*_0_ samples, we train all M base classifiers on these samples to obtain their initial weights $\beta_{0}$. Once the first batch of examples has been seen, new samples $\{(x_{i},y_{i}){\}}_{i=1}^{N_{k}}$ are classified using a weighted‐voting scheme. The prediction for each instance is given by


$$\begin{array}{c} {\hat{y}}_{\left(i\right)}=\text{arg}\max_{c}\sum_{m=1}^{M}V_{m}\;𝕀(f_{m}(x_{i})=c),\;i=1,\ldots ,N_{k}. \end{array}$$ (28)

where $f_{m}(x_{i})$ is the class‐vote (i.e., the output) of the m-th base classifier on input $x_{i}$, $V_{m}$ is the voting weight of the m-th base classifier in the current round. In the very first round of voting, we set $V_{m}=1$ for all classifiers. The final predicted label $\hat{y}$ is the class with the highest total vote. This weighted‐voting mechanism helps mitigate the large errors that might arise from a single classifier’s local bias.

To enable the ensemble classifier to better adapt to changes in the data stream – thus striking an effective balance between fully utilizing recent information and rapidly forgetting outdated knowledge – we adjust the class weights of the ensemble during the online learning phase. First, based on the concept drift detection result, we apply a forgetting factor λ to the entire ensemble model and incorporate it into the confusion matrix CM. This ensures that, when the new sample $\left(y_{k+1},\;{\hat{y}}_{k+1}\right)$ arrives in iteration k + 1, the matrix is updated in an exponentially decayed fashion, thereby emphasizing the impact of recent observations:


$$\begin{array}{c} CM(y_{k},\;{\hat{y}}_{k}{)}^{\prime }=\lambda \times CM(y_{k},\;{\hat{y}}_{k}). \end{array}$$ (29)

Next, in the decayed matrix, we increment by one the cell corresponding to the true label and predicted label of the current sample:


$$\begin{array}{c} CM(y_{k},\;{\hat{y}}_{k}{)}^{\prime }=CM(y_{k},\;{\hat{y}}_{k}{)}^{\prime }+1 \end{array}$$ (30)

We then update each base classifier’s voting weight $V_{m}$ according to its confusion‐matrix performance:


$$\begin{array}{c} V_{m}=\sqrt{\frac{TP_{m}}{TP_{m}+FN_{m}}\;\times \;\frac{TN_{m}}{TN_{m}+FP_{m}}} \end{array}$$ (31)

Because base classifiers vary in their sensitivity to the minority class, we introduce a class‐weight correction factor $CF_{m}$ for the minority‐class weight $w_{k}$:


$$\begin{array}{c} CF_{m}=\left\{ \begin{array}{cc} max(0.5,0.95\times CF_{m}) & \Delta k\geq \mu ,\\ min(2.0,1.05\times CF_{m}) & \Delta k<\mu , \end{array}\right. \end{array}$$ (32)

where $\Delta k=\frac{TP_{m}}{TP_{m}+FN_{m}}\;-\;\frac{TN_{m}}{TN_{,}m+FP_{m}}$. Finally, the updated weight of each base classifier:


$$\begin{array}{c} w_{k}=w_{k}\times CF_{m}. \end{array}$$ (33)

**Fig 4:**
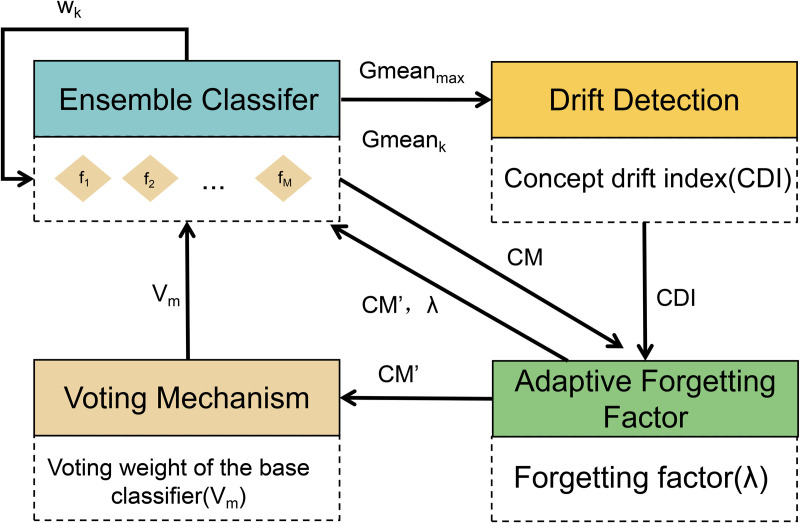
Flowchart of the adaptive update process.

The schematic of the complete update procedure is shown in Fig 4.

### Time complexity analysis

This subsection analyzes the computational complexity of EOS-PIELM from the perspective of time complexity. Since the proportion of data required in the initialization phase accounts for less than 3% of the total data in the experiments, and data streams are continuously generated, we mainly focus on the time complexity of the online learning phase.

For the standard OS-ELM, the per-instance computational cost mainly includes hidden layer output computation and recursive parameter updates, resulting in a time complexity of *O*(*dL* + *L*^2^). Therefore, for a data stream with *N* instances, the overall time complexity is *O*(*N*(*dL* + *L*^2^)).

In OS-PIELM, a pre-interference layer is introduced before the hidden layer, which adds an additional mapping cost of O(d·m), where *m* is the number of kernels. Since this term is typically dominated by *O*(*dL*), the overall per-instance complexity remains *O*(*dL* + *L*^2^). Let *T*_1_ denote the per-instance computational cost of OS-PIELM, i.e., $T_{1}=O(dL+L^{2})$. Therefore, OS-PIELM maintains the same order of time complexity as OS-ELM.

For the EOS-PIELM algorithm, an ensemble learning strategy with *M* base classifiers is adopted. Each classifier performs prediction and incremental updating, and their outputs are aggregated through a voting mechanism. Therefore, the total computational cost consists of three parts: the prediction and update cost of base classifiers, i.e., $M\times T_{1}$, the voting cost *O*(*M*), and the component update cost *O*(*M*). The overall time complexity can thus be expressed as $O((M\times T_{1}+M+M)\times N).$ Since the voting and component update operations mainly involve simple aggregation and bookkeeping, their computational costs are much smaller than that of model training and updating, i.e., $O(M)\ll M\times T_{1}$. Therefore, the overall time complexity of EOS-PIELM can be simplified as $O(M\times T_{1}\times N).$

Compared with the standard OS-ELM, EOS-PIELM increases the per-instance computational cost from *O*(*dL* + *L*^2^) to *O*(*M*(*dL* + *L*^2^)) due to the ensemble learning mechanism. However, since *M* is typically a small constant, the additional overhead grows linearly and remains computationally efficient. Therefore, EOS-PIELM achieves improved performance while maintaining a comparable computational complexity to OS-ELM.

## Experimental results and discussion

To evaluate the effectiveness of the algorithm, experiments were conducted on nine synthetic datasets and two real-time datasets. First, the sensitivity of the algorithm’s performance to its primary parameters was analyzed. Building on this analysis, the rationale and contributions of the algorithm’s designed components were further validated. Finally, the classification performance of EOS-PIELM was compared against other leading algorithms. All experiments were carried out on a Windows 10 machine with 8 GB of RAM and an Intel Core™ i5-9300 CPU.

### Datasets

The characteristics of 9 synthetic data streams and 2 real-time data streams are listed in Table 1. All synthetic data streams are generated by the Sea and Sine generators from the Scikit-Multiflow library, and their parameters can be customized.

(1) **Sine_IR generator** [55]**:** This dataset comprises a stream of binary classification synthetic data with 20000 samples, each sample has four features, the class label are binary, and in various experiments the imbalance ratio IR is set to 2, 4, or 9, with abrupt concept drift simulated by flipping the decision boundary at the sample indices 5000, 10000, and 15000, with the drift width set to 1, meaning that the change in the data distribution occurs instantaneously.
(2) **Sea_IR_T generator** [56]**:** This dataset is designed based on the SEA algorithm to simulate concept drift by dynamically adjusting decision thresholds. The resulting dataset contains 3 attributes (only 2 are relevant), and each stream consists of 20,000 instances with 3% noise added. We generate both abrupt-drift and gradual-drift streams, each containing 20,000 samples, with the imbalance ratio (IR) set to 2, 4, or 9. Concept drift occurs at sample indices 5000 and 15000. For the abrupt-drift setting (T = a), the drift width is set to 1, indicating that the change in the data distribution occurs instantaneously. For the gradual-drift setting (T = g), the drift width is set to 1000, meaning that the transition between concepts occurs progressively over a window of samples.
(3) **Sine_4_N generator** [55]**:** here N indicates the total number of samples; we generate datasets with N∈{20k,50k,100k,1M,5M} to evaluate algorithm performance on large-scale data, width is set to 1000, meaning that the transition between concepts occurs progressively over a window of samples.

**Table 1:** Dataset summary.

Dataset	Number of samples	Features	IR	Drift type
Sine_2	20K	4	2	abrupt, recurrent
Sine_4	20K	4	4	abrupt, recurrent
Sine_9	20K	4	9	abrupt, recurrent
Sea_2_a	20K	4	2	abrupt
Sea_4_a	20K	4	4	abrupt
Sea_9_a	20K	4	9	abrupt
Sea_2_g	20K	4	2	gradual
Sea_4_g	20K	4	4	gradual
Sea_9_g	20K	4	9	gradual
Sine_20K	20K	4	4	abrupt, recurrent
Sine_50K	50K	4	4	abrupt, recurrent
Sine_100K	100K	4	4	abrupt, recurrent
Sine_1M	1M	4	4	abrupt, recurrent
Sine_5M	5M	4	4	abrupt, recurrent
Weather	18159	8	Unknow	Unknow
Elec	45312	6	Unknow	Unknow

Considering that drift and imbalance ratio are Unknown in real-time data, this paper only introduces their basic characteristics.

(4) **Weather Dataset** [57]**:** This dataset contains weather information collected in Bellevue, Nebraska, from 1949 to 1999 and comprises 18159 instances. It includes eight relevant attributes, and the objective is to predict whether it will rain on a given date.
(5) **Elec Dataset** [57]**:** This is a widely used real‐world dataset in data‐stream learning. It consists of partial records from the New South Wales electricity market in Australia, spanning 1995–1998, and contains 45312 instances. The dataset includes six relevant attributes. Since electricity prices there fluctuate according to supply and demand rather than remaining fixed, the goal is to predict daily price movements (1 = increase, 0 = decrease).

### Comparison methods

In this study, we compare EOS-PIELM with six representative online learning algorithms, which are briefly described as follows:

- **WOS-ELM [24]:** A weighted OS-ELM approach that dynamically adjusts sample weights based on class distribution, improving minority-class recognition.
- **VWOS-ELM [25]:** VWOSELM is an improved OS-ELM method designed for imbalanced data stream classification. By introducing a weighting strategy, it increases the influence of minority-class samples during model updating, while the voting mechanism enhances the overall decision performance. In this way, VWOSELM alleviates the tendency of standard OS-ELM to favor majority classes. However, VWOSELM mainly focuses on class imbalance handling and does not explicitly model concept drift, which may limit its adaptability in dynamically evolving streaming environments.
- **FROS-ELM [21]**: The forgetting factor-based OS-ELM introduces a forgetting mechanism to gradually reduce the influence of outdated data, thereby enhancing the model’s adaptability to evolving data streams.
- **OS-ELM [26]:** The standard online sequential extreme learning machine that incrementally updates model parameters as new data arrives. It serves as a baseline for evaluating incremental learning performance.
- **LPP [58]:** Proposed by Elwell et al. (2011), LPP is an ensemble classifier designed for non-stationary environments (NSE). It utilizes a unique multi-classifier voting mechanism to incrementally learn from streaming data while adapting to concept drift.
- **SRP [55]:** Introduced by Gomes et al. (2019), SRP combines bagging and random subspace techniques for online ensemble learning. The default base learner is the Hoeffding tree, and the algorithm employs ADWIN for drift detection. SRP can also accommodate alternative base classifiers, providing flexibility for different streaming scenarios.

These models represent a diverse set of strategies for handling class imbalance, concept drift, and high-dimensional streaming data. By comparing EOS-PIELM against these methods, we aim to demonstrate the advantages of our proposed approach in simultaneously addressing both concept drift and class imbalance in dynamic data streams.

### Evaluation metrics

To quantitatively compare and analyze algorithm performance, this section describes the evaluation metrics used in experiments:

1. **Accuracy (Acc)**: Measures the proportion of correct predictions among all predictions, reflecting the model’s classification capability under current concepts.


$$\begin{array}{c} \text{Acc}=\frac{\text{TP}+\text{TN}}{\text{TP}+\text{TN}+\text{FP}+\text{FN}} \end{array}$$ (34)

2. **Recall Rate (Rec)**: Measures the proportion of correctly predicted positive samples among all actual positive samples, indicating the model’s coverage of minority class samples.


$$\begin{array}{c} \text{Rec}=\frac{\text{TP}}{\text{TP}+\text{FN}} \end{array}$$ (35)

3. **Specificity (Spe)**: Measures the proportion of correctly identified negative samples among all actual negative samples, reflecting the model’s ability to exclude *majority class* samples.


$$\begin{array}{c} \text{Spe}=\frac{\text{TN}}{\text{TN}+\text{FP}} \end{array}$$ (36)

4. **Gmean**: Represents the geometric mean of Recall and Specificity, indicating the model’s balanced performance across both classes.


$$\begin{array}{c} \text{Gmean}=\sqrt{\text{Rec}\times \text{Spe}} \end{array}$$ (37)

5. **Classification Distance D(Rec, Spe)**: Measures the absolute difference between Recall and Specificity, quantifying the model’s bias in class-wise recognition capability.


$$\begin{array}{c} D(\text{Rec},\text{Spe})=|\text{Rec}-\text{Spe}| \end{array}$$ (38)

For classification tasks, accuracy (Acc) is the most widely used performance metric, as it measures how well an algorithm labels the overall sample set. However, when a data stream exhibits class imbalance, accuracy is no longer an ideal indicator. The Gmean [59] metric proposed by Kubat and colleagues captures a classifier’s overall performance and is considered the most important measure for imbalanced data stream classification. Therefore, in our experiments, greater emphasis is placed on Gmean performance.

### Analyzing the sensitivity of parameters

The classification performance of a data stream with concept drift and class imbalance mainly depends on two parameters: the block size $N_{k}$ and the imbalance‐change threshold μ. We select these parameters from the following ranges: $N_{k}\in \{50,\;100,\;125,\;150,\;175,\;200\},\;\mu \in \{0.01,\;0.05,\;\;0.1,\;0.15,\ldots ,\;0.50\}.$ According to the report of the previous literature [60], we set the default $N_{k}=100$ and then conduct a series of experiments (for different μ values) on all datasets except Sine_4_N.

**Fig 5:**
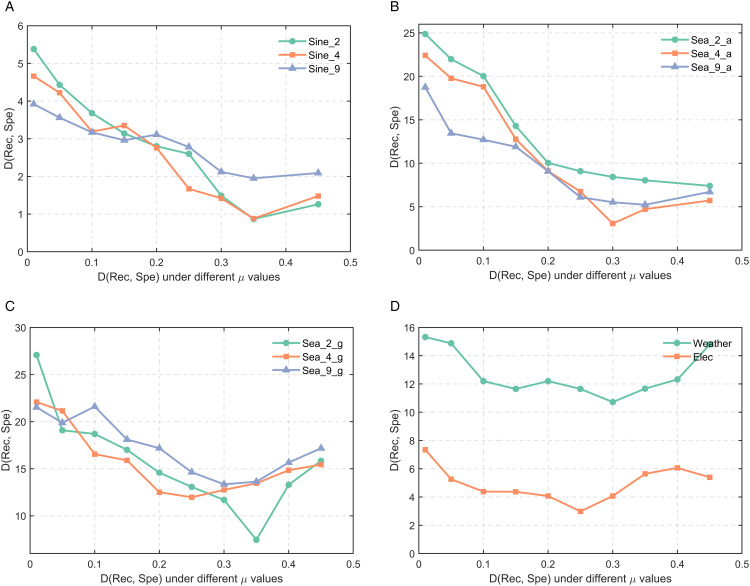
D(Rec,Spe) of EOS-PIELM under different μ for synthetic and real-time data streams. (a) SINE, (b) SEA_Abrupt, (c) SEA_Gradual, (d) Weather-Elec.

**Fig 6:**
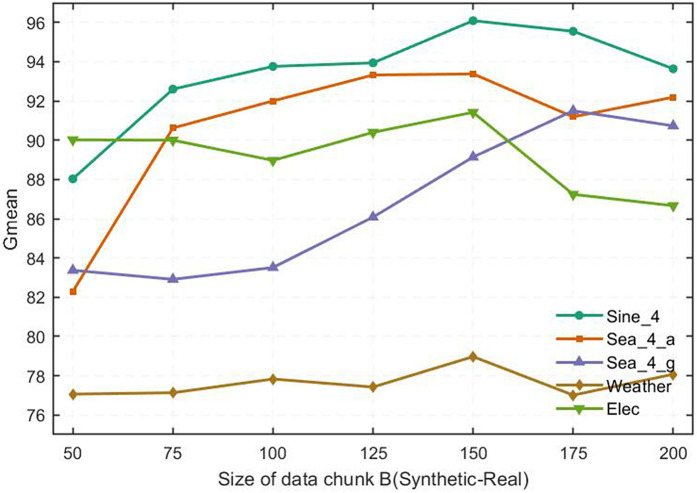
Gmean of EOS-PIELM under different sizes of data chunks for synthetic and real-time data streams.

From the Fig 5, As shown in Fig 5, the parameter μ significantly influences the stability–adaptivity trade-off of EOS-PIELM. When μ<0.1, the model becomes overly sensitive to minor fluctuations in class distribution, leading to large oscillations in D(Rec,Spe). Conversely, when μ>0.35, the model reacts too slowly to real imbalance changes, resulting in delayed adaptation. Notably, the interval μ∈[0.1,0.35] yields consistently lower D(Rec,Spe) across both synthetic and real datasets, indicating a balanced performance between minority and majority classes. As shown in Fig 6, $N_{k}$ increases, the Gmean initially improves due to reduced variance and more reliable parameter estimation. However, beyond a certain point (approximately $N_{k}$ = 150), the Gmean begins to decline. This is because excessively large blocks reduce the update frequency, weakening the model’s responsiveness to concept drift.

The optimal combinations of parameters on 11 data streams are obtained as follows:

**Table:** 

Sine_2: $\begin{array}{c} N_{k}=150 \end{array}$, $\begin{array}{c} \mu =0.35 \end{array}$ Sine_4: $\begin{array}{c} N_{k}=150 \end{array}$, $\begin{array}{c} \mu =0.35 \end{array}$ Sine_9: $\begin{array}{c} N_{k}=150 \end{array}$, $\begin{array}{c} \mu =0.35 \end{array}$
Sea_2_a: $N_{k}=125$, μ=0.25 Sea_4_a: $N_{k}=125$, μ=0.30 Sea_9_a: $N_{k}=125$, μ=0.35
Sea_2_g: $N_{k}=175$, μ=0.35 Sea_4_g: $N_{k}=175$, μ=0.25 Sea_9_g: $N_{k}=175$, μ=0.30
Weather: $N_{k}=150$, μ=0.30 Elec: $N_{k}=150$, μ=0.30

### Analyzing contribution of components

To assess how the pre-interference layer in EOS-PIELM enhances both classification performance and concept drift detection, we ran experiments on all datasets except Sine_4_N. Keeping every other condition constant, we then replaced EOS-PIELM’s OS-PIELM base classifier with OS-ELM renaming the resulting model EOS-ELM and compared their results.

**Fig 7:**
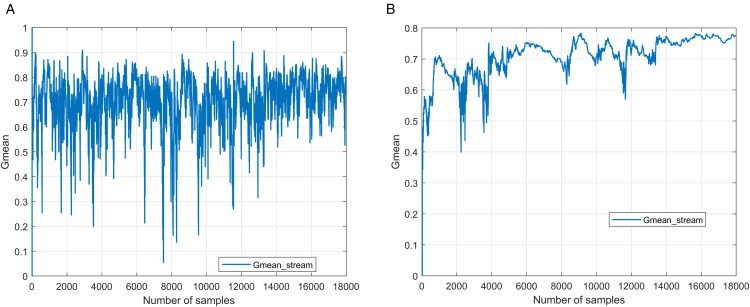
Time-wise Gmean Comparison between EOS-PIELM and EOS-ELM. [a] Result of EOS-ELM. [b] Result of EOS-PIELM.

The experimental results listed in Table 2 show that, on both synthetic and real datasets, the pre-interference layer brings considerable improvements in classification performance. In particular, the relatively high Gmean values indicate that introducing the pre-interference layer enhances the algorithm’s adaptability to imbalanced data streams and to those undergoing concept drift. By comparing classification performance on synthetic and real datasets under identical conditions, EOS-PIELM exhibits notably superior results when handling high-dimensional and complex data. Furthermore, taking the Weather dataset from the real data as an example, we present its real-time Gmean variation plot. From Fig 7, EOS-PIELM exhibits faster recovery after drift points and smaller performance degradation than EOS-ELM. This indicates that the proposed Gmean-based drift detector and adaptive forgetting mechanism enable the model to rapidly adapt to distribution changes while preserving stable classification performance. in terms of time-wise Gmean. In particular, at drift points, EOS-PIELM exhibits faster recovery and smaller performance degradation, indicating its superior adaptability to abrupt distribution changes. Furthermore, the fluctuations in Gmean are significantly reduced, demonstrating improved stability under noisy and imbalanced conditions. These results confirm that the proposed interference layer, combined with adaptive forgetting and dynamic weighting mechanisms, effectively enhances both drift detection and class imbalance handling.

**Table 2:** Comparison of EOS-PIELM and EOS-ELM.

Number	Dataset	EOS-PIELM		EOS-ELM	
		Acc	Gmean	Acc	Gmean
1	Sine_2	91.47	95.22	85.03	91.51
2	Sine_4	90.09	96.09	86.08	92.93
3	Sine_9	91.54	96.37	85.63	92.86
4	Sea_2_a	91.47	92.24	90.74	85.31
5	Sea_4_a	90.09	92.00	92.03	85.48
6	Sea_9_a	91.54	93.73	91.07	86.26
7	Sea_2_g	93.34	91.93	94.69	82.02
8	Sea_4_g	94.82	91.50	93.81	79.30
9	Sea_9_g	94.51	91.16	94.12	80.16
10	Weather	79.74	78.97	78.83	73.19
11	Elec	82.88	91.42	76.09	84.84

### Comparison with other methods

As shown in Table 3 and 4, our proposed EOS-PIELM method outperforms the other six online classifiers: VWOS-ELM [25], WOS-ELM [24], FROS-ELM [61], OS-ELM [26], LPP [58], and SRP [55] in most data streams. We evaluated all algorithms using six performance metrics (Accuracy, Recall, Specificity, Gmean and D(Rec, Spe)), and EOS-PIELM consistently achieved the highest Gmean across most datasets. Since Gmean simultaneously evaluates the recognition performance of majority and minority classes, the superior Gmean values indicate that the proposed dynamic weighting strategy effectively alleviates the bias toward majority classes and improves the classification performance of minority-class samples under imbalanced data streams. Furthermore, EOS-PIELM obtained the smallest D(Rec,Spe) values in most datasets. This demonstrates that the proposed method maintains a more balanced recognition capability between majority and minority classes, further confirming its effectiveness in handling class imbalance.

**Table 3:** Performance Comparison of all methods.

Dataset	Evaluation	VWOS-ELM	WOS-ELM	FROS-ELM	OS-ELM	LPP	SRP	EOS-PIELM
Sine_2	Acc	48.05	47.89	**91.78**	67.59	90.14	78.99	83.93
	Rec	44.28	42.95	**97.38**	90.92	92.08	92.49	95.65
	Spe	55.58	57.75	80.59	20.96	86.25	52.00	**94.78**
	Gmean	49.61	49.80	88.59	43.65	89.12	69.35	**95.22**
	D(Rec,Spe)	11.30	14.80	16.79	69.96	5.83	40.49	**0.87**
Sine_4	Acc	44.89	44.14	**92.44**	80.72	92.05	84.55	84.03
	Rec	41.49	39.99	**98.97**	96.76	94.69	97.41	95.65
	Spe	85.48	60.76	66.26	16.47	81.45	33.05	**96.53**
	Gmean	49.26	49.29	80.98	39.92	87.82	56.74	**96.09**
	D(Rec,Spe)	16.99	20.77	32.71	80.29	13.24	64.36	**0.88**
Sine_9	Acc	40.82	39.45	94.32	90.28	**94.49**	91.23	86.88
	Rec	38.24	36.49	**99.79**	99.22	96.91	99.48	97.41
	Spe	63.82	65.80	45.50	10.43	72.88	17.61	**95.34**
	Gmean	49.40	49.00	67.38	32.17	84.04	41.86	**96.37**
	D(Rec,Spe)	25.58	29.31	54.29	88.79	24.03	81.87	**2.07**
Sea_2_a	Acc	90.16	90.53	90.28	88.71	90.15	90.76	**91.47**
	Rec	93.66	93.59	97.51	**98.03**	93.24	95.67	88.61
	Spe	83.11	84.36	75.70	69.92	83.93	80.88	**96.01**
	Gmean	88.23	88.86	85.92	82.79	88.46	87.96	**92.24**
	D(Rec,Spe)	10.55	9.23	21.81	82.11	9.31	14.79	**7.40**
Sea_4_a	Acc	91.28	90.82	**91.69**	91.24	91.56	90.15	90.09
	Rec	93.02	91.95	98.57	**98.74**	95.15	97.68	90.49
	Spe	84.31	86.29	64.06	61.10	77.13	59.89	**93.54**
	Gmean	88.56	89.08	79.46	77.67	85.67	76.49	**92.00**
	D(Rec,Spe)	8.71	5.66	34.51	37.64	18.02	37.79	**3.06**
Sea_9_a	Acc	90.34	87.68	**94.30**	94.08	93.47	92.18	91.54
	Rec	90.86	87.63	98.91	98.95	96.77	**99.44**	96.58
	Spe	85.68	88.19	52.72	50.21	63.71	26.85	**91.16**
	Gmean	88.23	87.91	72.21	70.49	78.52	51.67	**93.73**
	D(Rec,Spe)	5.18	**0.56**	46.19	48.74	33.06	72.59	5.22

**Table 4:** Performance Comparison of all methods.

Dataset	Evaluation	VWOS-ELM	WOS-ELM	FROS-ELM	OS-ELM	LPP	SRP	EOS-PIELM
Sea_2_g	Acc	89.77	90.07	89.84	88.63	89.34	83.64	**93.34**
	Rec	93.36	92.66	97.17	**97.94**	92.49	93.14	95.73
	Spe	82.63	84.90	75.24	70.09	83.09	64.73	**88.28**
	Gmean	87.83	88.70	85.50	82.85	87.66	77.65	**91.93**
	D(Rec,Spe)	10.73	7.76	21.93	27.85	9.40	28.41	**7.45**
Sea_4_g	Acc	90.68	90.02	91.48	90.97	90.89	86.64	**94.82**
	Rec	92.40	90.93	98.48	**98.76**	94.80	98.09	97.68
	Spe	83.56	**86.24**	62.62	58.89	74.78	39.43	85.71
	Gmean	87.87	88.55	78.53	76.26	84.20	62.19	**91.50**
	D(Rec,Spe)	8.84	**4.69**	35.86	39.87	20.02	58.66	11.97
Sea_9_g	Acc	89.01	87.38	94.01	93.81	92.65	91.83	**94.51**
	Rec	89.56	87.40	98.76	98.95	96.23	**99.51**	98.08
	Spe	84.10	**87.24**	51.80	48.15	60.91	23.59	84.73
	Gmean	86.79	87.32	71.52	69.02	76.56	48.45	**91.16**
	D(Rec,Spe)	5.46	**0.16**	46.96	50.80	35.32	75.92	13.35
Weather	Acc	72.20	70.94	78.81	76.76	69.43	75.38	**79.74**
	Rec	85.43	**86.53**	59.72	62.19	54.33	54.99	73.80
	Spe	66.11	63.77	**87.59**	83.46	76.38	84.75	84.51
	Gmean	75.15	74.28	72.32	72.04	64.42	68.27	**78.97**
	D(Rec,Spe)	19.32	22.76	27.87	21.27	22.05	29.76	**10.72**
Elec	Acc	75.84	75.17	**84.63**	75.01	75.08	84.29	82.88
	Rec	62.43	63.54	78.03	54.26	69.49	78.02	**90.51**
	Spe	85.71	83.74	89.50	90.30	79.19	88.91	**92.35**
	Gmean	73.15	72.94	83.57	70.00	74.18	83.29	**91.42**
	D(Rec,Spe)	23.28	20.20	11.47	36.04	9.70	10.89	**1.84**
	p-value		$1.20\times 10^{-7}$(Gmean)		$2.21\times 10^{-8}$(D(Rec,Spe))			

On nine synthetic datasets (Sine_IR, Sea_IR_a, and Sea_IR_g among them), four algorithms: FROS-ELM, OS-ELM, LPP, and SRP exhibited rapidly declining minority-class accuracy (Specificity) as the imbalance ratio (IR) increased. In those scenarios, D(Rec, Spe) widened substantially and Gmean dropped sharply, indicating that these four methods lack the ability to adapt to class imbalance. By contrast, EOS-PIELM demonstrated a significant advantage on the Sine datasets in both Gmean and D(Rec, Spe). This illustrates that our incorporation of concept-drift detection and minority-class weighting enables the classifier to react quickly to abrupt concept drifts and adjust its model in a timely manner. On the Sea datasets, EOS-PIELM maintained strong minority-class accuracy and achieved superior Gmean values compared to the other algorithms. These results confirm that EOS-PIELM can effectively handle both gradual and abrupt concept drifts in streaming data.

Based on the statistical analysis framework, we employed the Friedman test to evaluate the experimental results in terms of Gmean and D(Rec,Spe). The p-value for Gmean is $1.20\times 10^{-7}$, and the p-value for D(Rec,Spe) is $2.21\times 10^{-8}$. The statistical results confirm that there are significant differences among all the compared algorithms. To further evaluate the statistical significance of the performance improvements, the Wilcoxon signed-rank test was conducted across multiple datasets, as shown in Table 5. The results indicate that the proposed EOS-PIELM achieves statistically significant improvements over most baseline methods in terms of both Gmean and D(Rec,Spe). Specifically, for VWOS-ELM, EOS-PIELM shows significant improvement in both metrics (*p* < 0.05), demonstrating its superior capability in handling imbalanced data streams. In comparison with FROS-ELM, OS-ELM, LPP, and SRP, the proposed method achieves highly significant improvements, indicating its strong and consistent performance advantages across different datasets. For WOS-ELM, EOS-PIELM exhibits statistically significant improvement in Gmean, while the difference in D(Rec,Spe) is not statistically significant. This suggests that although the two methods have comparable performance in terms of class-wise balance consistency, EOS-PIELM provides more stable and reliable classification performance overall. Overall, the statistical analysis confirms that EOS-PIELM not only improves the average performance but also achieves consistent and statistically significant superiority across multiple data stream scenarios, validating its effectiveness in addressing class imbalance and concept drift simultaneously.

**Table 5:** Wilcoxon signed-rank test results of EOS-PIELM against baseline methods.

Comparison	p-value (Gmean)	p-value (D(Rec,Spe))	Significance(α=0.05)
VWOS-ELM	< 0.001	0.0322	Significant in both metrics
WOS-ELM	< 0.001	0.1475	Significant only in Gmean
FROS-ELM	< 0.001	< 0.001	Significant in both metrics
OS-ELM	< 0.001	< 0.001	Significant in both metrics
LPP	< 0.001	< 0.001	Significant in both metrics
SRP	< 0.001	< 0.001	Significant in both metrics

Dealing with complex, real-time data streams is one of the biggest challenges in online classification. On the Weather and Elec real-time datasets, EOS-PIELM again performed very well: its Gmean values were at least 5–10 percentage points higher than those of the competing algorithms. This demonstrates that EOS-PIELM can maintain robust classification performance in complex, real-time streams. Moreover, as shown in Table 6, EOS-PIELM remains stable and continues to deliver excellent performance even when confronted with large-scale datasets.

**Table 6:** Performance of EOS-PIELM in large-scale datasets.

Metric	Sine_4_20K	Sine_4_50K	Sine_4_100K	Sine_4_1M	Sine_4_5M
Acc	84.03	92.15	94.15	96.36	96.47
Rec	95.65	96.98	96.23	96.76	95.16
Spe	96.53	95.96	98.73	95.11	99.00
Gmean	96.09	96.47	97.47	95.93	97.06
D(Rec,Spe)	0.88	1.02	2.50	1.66	3.83

## Conclusion

In data streams, class imbalance often coexists with concept drift, posing significant challenges for traditional online classifiers. To address this, we propose an online ensemble learning algorithm that is robust to both drift and imbalance. First, we introduce an OS-PIELM network architecture: by augmenting the standard OS-ELM with a nonlinear pre-interference layer, incoming sequential samples are mapped into a more complex feature space, which in turn enhances the performance of the downstream cascade classifier. Building on this base, we then develop an adaptive ensemble framework that integrates a forgetting factor, concept drift detection, and a dynamic weighting strategy to handle both emerging drifts and skewed class distributions in real time. Finally, experiments on eleven benchmark streams each exhibiting both class imbalance and various types of drift demonstrate that our method adapts quickly to changing concepts while maintaining robustness against imbalance.

Although EOS-PIELM delivers strong results under these challenging conditions, its ability to detect and adapt to *virtual drift* still warrants further investigation. Virtual drift refers to a situation in which the input feature distribution *P*(*X*) changes while the conditional distribution *P*(*Y*|*X*) remains unchanged, meaning that the decision boundary does not vary. Although this type of drift does not immediately influence classification accuracy, it may gradually reduce the robustness of the classifier if not properly handled.

Moreover, since many practical applications involve multiclass scenarios, future work will extend our framework to address concept drift and class imbalance in multi-stream environments, with potential applications in face recognition and sentiment analysis.
