# Supplementary material for: Ensemble learning-based online sequential pre-interference extreme learning for concept drifting and class imbalanced data streams
Source: PLoS One. 2026 Jul 31;21(7):e0353728. doi: 10.1371/journal.pone.0353728 (PMC13426951; doi:10.1371/journal.pone.0353728)
Supplement: Attachment — Submitted filename: Response_PONE-D-25-66864.docx [file pone.0353728.s002.docx]

Dear Dr. Wang,

Thank you for your email and for outlining the additional requirements for our manuscript. We have carefully addressed all the points raised and revised our submission accordingly. Our responses are detailed below:

**Question 1：**Please ensure that your manuscript meets PLOS ONE's style requirements, including those for file naming. The PLOS ONE style templates can be found at
[https://journals.plos.org/plosone/s/file?id=wjVg/PLOSOne_formatting_sample_main_body.pdf](https://journals.plos.org/plosone/s/file?id=wjVg/PLOSOne_formatting_sample_main_body.pdf) and [https://journals.plos.org/plosone/s/file?id=ba62/PLOSOne_formatting_sample_title_authors_affiliations.pdf](https://journals.plos.org/plosone/s/file?id=ba62/PLOSOne_formatting_sample_title_authors_affiliations.pdf)

**Response:**We have revised the manuscript to ensure full compliance with PLOS ONE formatting guidelines, including file structure, organization, and naming conventions. The manuscript has been updated using the provided templates to meet all journal requirements.

**Question 2:**Please note that PLOS One has specific guidelines on code sharing for submissions in which author-generated code underpins the findings in the manuscript. In these cases, we expect all author-generated code to be made available without restrictions upon publication of the work. Please review our guidelines at https://journals.plos.org/plosone/s/materials-and-software-sharing#loc-sharing-code and ensure that your code is shared in a way that follows best practice and facilitates reproducibility and reuse.

**Response:**We thank the editor for this comment. In accordance with PLOS ONE’s code sharing guidelines, we have made the source code used in this study publicly available without restriction. The code is accessible at the following repository:

https://github.com/cutainrob/EOS-PIELM

This repository provides the implementation of the proposed methods and is intended to facilitate transparency, reproducibility, and reuse of our work.

**Question 3:**Please provide a complete Data Availability Statement in the submission form, ensuring you include all necessary access information or a reason for why you are unable to make your data freely accessible. If your research concerns only data provided within your submission, please write "All data are in the manuscript and/or supporting information files" as your Data Availability Statement.

**Response:**The data used in this study consist of artificially generated datasets and publicly available datasets from the UCI Machine Learning Repository. The specific sources of all datasets have been cited in the manuscript. Accordingly, we have included the following Data Availability Statement in the revised manuscript:

“All data underlying the findings in this study are fully available without restriction. The artificial datasets were generated according to the procedures described in the manuscript. The public datasets were obtained from the UCI Machine Learning Repository, and their specific sources are cited in the main text.”

**Question 4:**Thank you for stating the following financial disclosure:

This work is supported by the Fujian Provincial Natural Science Foundation Project (2023J011015, 2022J011179, 2024J01888), Ministry of Education Industry-Academia Collaborative Education Project(231102311285117), Putian Science and Technology Plan Project(2024NJJ009, 2023GJGZ003,2024GZ2001PTXY17), Putian University Graduate Research and Innovation Project(yjs2024054), Fujian Province Science and Technology Special Envoy Project(F2022KTP027, F2024KTP086), National Natural Science Foundation of China (grant number 52577115), Putian High end Equipment Industry Technology Research Institute(2023GJGZ002). Please state what role the funders took in the study. If the funders had no role, please state: "The funders had no role in study design, data collection and analysis, decision to publish, or preparation of the manuscript."

**Response:**We thank the editor for this comment. We have clarified the role of the funders in both the manuscript and the cover letter. The statement is as follows:

“The funders had no role in study design, data collection and analysis, decision to publish, or preparation of the manuscript.”

**Question 5:**We note that you have provided funding information that is not currently declared in your Funding Statement. However, funding information should not appear in the Acknowledgments section or other areas of your manuscript. We will only publish funding information present in the Funding Statement section of the online submission form.

**Response:**We thank the editor for this clarification. We have carefully revised the manuscript and removed all funding information from the Acknowledgments section and any other parts of the main text. The funding information is now provided exclusively in the Funding Statement section of the online submission form, in full compliance with PLOS ONE requirements.

**Question 6:**Please amend your list of authors on the manuscript to ensure that each author is linked to an affiliation. Authors’ affiliations should reflect the institution where the work was done (if authors moved subsequently, you can also list the new affiliation stating “current affiliation:….” as necessary).

**Response:**We thank the editor for this comment. We have carefully reviewed the author list and affiliations in the manuscript. Each author is correctly linked to their respective affiliation, and the affiliations accurately reflect the institutions where the work was conducted. No inconsistencies were identified, and the formatting has been checked to ensure compliance with the journal’s requirements.

**Question 7:**Please include a caption for figure 1.

**Response:**We thank the editor for this comment. We have added a caption for Figure 1 in the revised manuscript. The figure caption has been included in the main text below the figure, in accordance with the journal’s formatting requirements.

**Question 8:**We note that Figure(s) 1-4 in your submission contain copyrighted images. All PLOS content is published under the Creative Commons Attribution License (CC BY 4.0), which means that the manuscript, images, and Supporting Information files will be freely available online, and any third party is permitted to access, download, copy, distribute, and use these materials in any way, even commercially, with proper attribution. For more information, see our copyright guidelines: <http://journals.plos.org/plosone/s/licenses-and-copyright.>

**Response:**We thank the editor for this comment. We confirm that Figures 1–4 in the manuscript are original figures created by the authors. They do not contain any copyrighted material from third-party sources. Therefore, they are fully compliant with the Creative Commons Attribution License (CC BY 4.0) requirements of PLOS ONE.

Reviewer #1:

**Question 1：**What is the relationship between the proposed concept drift and class imbalance? Is it the result of problem A + problem B, or an intersection of problem A and problem B? This needs to be explained in detail.

**Response:**Thank you for this valuable comment. We agree that the relationship between concept drift and class imbalance was not sufficiently clarified in the original manuscript. In the revised version, we have improved the corresponding description to explicitly explain their relationship.

First, we introduce a practical bearing fault diagnosis scenario to illustrate that both concept drift and class imbalance commonly occur in data streams. Specifically, variations in operating conditions lead to dynamically changing data distributions, resulting in concept drift, while the scarcity of fault samples in early stages leads to class imbalance. More importantly, we clarify that these two issues are not independent but inherently coupled. Concept drift can alter class prior probabilities or class-conditional distributions, thereby causing the degree of class imbalance to evolve dynamically over time. Conversely, class imbalance further increases the difficulty of learning in drifting data streams, particularly by degrading the recognition performance of minority classes, and may adversely affect online learning models, including concept drift detection mechanisms. Accordingly, we emphasize that the problem addressed in this work is not a simple combination of two independent challenges, but a coupled learning problem under both concept drift and class imbalance. The revised manuscript has been updated to reflect this clarification.

**Question 2:**Supplement the description of Fig. 3 (Problem: The descriptions of different figures are completely identical).

**Response:**We fully agree with your suggestion. To enhance the interpretability of Figure 3, we have added the following explanation in the first paragraph of the OS-PIELM subsection:

“As shown in Fig. 3, the upper subfigure illustrates a pre-interference layer with two nodes, while the lower subfigure illustrates a pre-interference layer with three nodes. The orange rectangles and green circles denote two different classes of samples. After nonlinear kernel mapping of the original samples, the samples become more separable in the transformed feature space, indicating that the proposed interference layer enhances the discriminative representation of the data.” This revision has been incorporated into the revised manuscript.

**Question 3:**The ordinate of Fig. 6 does not match the description.

Response:We appreciate your careful review and constructive feedback. Upon verification, we found that there was a labeling error in the vertical axis of Figure 6. We have replaced the original figure, corrected the vertical axis label by changing “G-Mean” to “Gmean,” and revised the figure caption accordingly: Fig 6. Gmean of EOS-PIELM under different under different sizes of data chunks for synthetic and real-time data streams

**Question 4：**The punctuation in "comparison among and other methods" is incorrect.

**Response:**Thank you for your careful review. We agree that the expression “comparison among and other methods” in the title is grammatically incorrect. It has been revised to “Comparison with other methods”to ensure clarity and correctness. The revision has been incorporated into the manuscript.

**Question 5：**Supplement the description of the comparative model

**Response:**Thank you for the valuable comment. We agree that a clearer description of the comparative models can help readers better understand their characteristics and the rationale for selecting them as baselines. In the revised manuscript, we have added brief descriptions of all comparative methods, including VWOS-ELM, WOS-ELM, FROS-ELM, OS-ELM, LPP, and SRP, to clarify their main mechanisms and application scenarios. The corresponding descriptions have been added in Comparison methods.

**Question 6：**Add an explanation of "virtual drift," etc.

**Response:**We sincerely thank the reviewer for the valuable suggestion. We agree that providing a clearer explanation of “virtual drift” can improve readers’ understanding of this concept. In the revised manuscript, we have added a more detailed description of virtual drift and further clarified the distinction between virtual drift and real concept drift.

**Question 7:**Supplement the width of the drift.

**Response:**We sincerely thank the reviewer for the valuable suggestion. We agree that explicitly specifying the drift width in the data stream experimental settings helps to more clearly characterize changes in data distribution. In the revised manuscript, we have added a detailed description of the drift width in the Sine_IR data generator. Since this dataset simulates abrupt concept drift by directly flipping the decision boundary, the drift width is set to 1, indicating that the change in data distribution occurs instantaneously. For the Sea_IR_T data generator, the drift width is set to 1 for abrupt concept drift, while it is set to 1000 for gradual drift, indicating that the data distribution transitions progressively over a certain interval. The corresponding explanations have been added to the “Datasets” subsection under “Experimental Results and Discussion” in the revised manuscript.

**Question 8:**The authors need to supplement the discussion of the broader field of stream learning, particularly regarding the current state of research in online learning, and point out the connections and differences between the proposed method and related works to better illustrate the work presented in this paper.

**Response:**Thank you for your valuable comment. We agree that a clearer discussion of the broader field of stream learning, as well as the connections and differences between related works and the proposed method, would improve the clarity and completeness of the manuscript.

In the revised manuscript, we have expanded the introduction to include a more comprehensive review of recent advances in online learning for data streams. Specifically, we discuss representative methods addressing different challenges, including incomplete or uncertain data (e.g., OLIFL), concept drift adaptation, and semi-supervised learning. For each category, we not only describe the main ideas and advantages but also explicitly analyze their limitations. Furthermore, we have clarified the connections and differences between these existing approaches and the proposed method. The revised discussion highlights that most existing methods focus on a single aspect of data stream learning and lack the ability to simultaneously address multiple interacting challenges, particularly the joint presence of concept drift and class imbalance. Based on this analysis, we explicitly identify the research gap and motivate the proposed EOS-PIELM framework, which integrates concept drift detection, adaptive forgetting mechanisms, and class imbalance handling into a unified online learning framework.

These revisions have been incorporated into the introduction section of the manuscript to improve its logical coherence and to better position the contributions of this work within the broader research landscape.

Reviewer #2

**Question 1：**The key terms pre-interference layer and interference layer are used interchangeably throughout the manuscript to refer to the same structural component added to the OS-ELM model. Additionally, the concept of class imbalance is described with two inconsistent expressions: class imbalanced and category imbalance. Such terminological inconsistency violates the basic norms of academic writing and may cause confusion for readers in understanding the core design and research object of the study.

**Response:**Thank you for the careful observation. We agree that consistent terminology is essential for clarity in academic writing. In the revised manuscript, we have unified the terminology throughout the paper. Specifically, the term “pre-interference layer” is consistently used to refer to the structural component inserted between the input and hidden layers of the OS-ELM model. In addition, the expression “class imbalance” is used uniformly to describe the imbalance problem in data streams. All inconsistent terms have been corrected accordingly in the revised manuscript.

**Question 2：**Grammatical Errors and Inadequate Proofreading：The manuscript contains multiple grammatical errors and spelling mistakes, which reflect a lack of careful proofreading. For example, the conjunction redundancy in the sentence "Although the interference layer... but the class labels..." (the simultaneous use of Although and but violates English grammatical rules); spelling errors such as Performce (correct: Performance) and ndr rves (correct: curves) in the text and figure captions. These issues significantly reduce the readability and formality of the manuscript.

**Response:**Thank you for the careful review and for pointing out these language issues. We agree that grammatical accuracy and consistent proofreading are essential for maintaining the clarity and readability of the manuscript. In the revised version, we have carefully proofread the entire manuscript and corrected the grammatical and spelling errors identified by the reviewer. Specifically, redundant conjunctions such as the simultaneous use of “Although” and “but” have been corrected, and spelling errors in both the main text and figure captions (e.g., “Performancece” and “ndr rves”) have been fixed. In addition, the manuscript has been thoroughly checked to ensure linguistic accuracy and consistency throughout the paper.

**Question 3:**Insufficient Interpretation of Key Figures: Key experimental figures (Figure 5, Figure 6, Figure 7) are only briefly referenced with a general conclusion in the text, without detailed data-driven interpretation and analysis. The manuscript fails to elaborate on the specific trends, critical inflection points, and statistical characteristics of the experimental results presented in these figures, nor does it link the figure data to the core research hypotheses and algorithm performance conclusions of the study. This makes the experimental results lack sufficient empirical support and weakens the persuasiveness of the research findings.

**Response:**We sincerely thank the reviewer for the insightful comment. We agree that a more detailed, data-driven interpretation of the key experimental figures would strengthen the empirical support and overall persuasiveness of the manuscript. In the revised version, we have supplemented the analysis in the parameter sensitivity analysis section and analyzing contribution of components section, where we provide a more comprehensive interpretation of Figures 5, 6, and 7. Specifically, we elaborate on the observed trends, key inflection points, and relevant statistical characteristics, and further relate these observations to the core research hypotheses and the performance of the proposed algorithm.

**Question 4:**Superficial Review of Existing Literature and Unclear Research Gaps: The review of existing research merely lists the improved methods of OS-ELM for data stream learning in a descriptive manner, without systematically sorting out and summarizing the core research challenges in the field of class-imbalanced data stream learning with concept drift (e.g., poor coupling of multiple functional modules, inadequate handling of multi-class classification scenarios, weak noise resistance of algorithms). More importantly, the manuscript does not explicitly establish the corresponding relationship between the identified research gaps and the innovative points of the proposed EOS-PIELM algorithm, which makes it difficult for readers to recognize the theoretical and practical contributions of this study relative to the existing literature.

**Response:**Thank you for your valuable comment. We agree that, in the original manuscript, the core research challenges in class-imbalanced data stream learning with concept drift were not clearly summarized in a structured manner, and the relationship between these research gaps and the proposed method was not explicitly established.

In the revised manuscript, we have made systematic revisions to address this issue (the newly added content is highlighted in the revised manuscript in the manuscript):

First, we have added a new paragraph to systematically summarize the key research challenges in this field, including: (1) insufficient coupling among functional modules, (2) unreliable drift detection under class imbalance, and (3) difficulty in modeling dynamic and unstable data distributions. This part is presented in a structured (itemized) form in the Introduction (highlighted in purple), providing a problem-driven summary of the limitations of existing methods. Second, in the related work review and problem analysis sections (highlighted in purple), we have further supplemented and strengthened the analysis of the limitations of existing methods. Specifically, regarding the issue that traditional error-rate-based drift detection methods may fail under class imbalance, we have added a detailed mechanism-level explanation, indicating that the dominance of majority-class samples may mask the misclassification of minority-class instances, thereby reducing detection reliability. In addition, we have elaborated on the impact of temporal variability and weak correlations in data streams on model stability. Furthermore, we have revised the introduction and contribution sections of the proposed method (highlighted in purple) to explicitly establish the correspondence between the identified challenges and the components of the EOS-PIELM algorithm. Specifically, the proposed ensemble framework and integrated design are intended to improve module coupling; the Gmean-based drift detection method combined with an adaptive forgetting mechanism enhances detection reliability under class imbalance; and the nonlinear mapping layer together with the adaptive learning strategy improves robustness under dynamic and unstable data distributions.

Through these revisions, we have not only provided a systematic summary of the research challenges, but also clearly established the relationship between the identified research gaps and the contributions of the proposed method, thereby improving the overall logical coherence and clarity of the manuscript.

Reviewer #3:

**Question 1:**Clarify the Role of the Pre-Interference Layer: The pre-interference layer is a key contribution. However, its theoretical motivation could be elaborated further: Is φ(x) fixed or randomly initialized? How does this differ from standard kernel ELM or random feature mapping? Does it increase representational capacity theoretically? A brief discussion on its theoretical interpretation would enhance clarity.

**Response:**We sincerely thank the reviewer for the valuable comment. We agree that a clearer theoretical explanation of the pre-interference layer would improve the overall clarity of the manuscript. In the revised version, we have further clarified the mechanism of the pre-interference layer, including its formulation and implementation details. In addition, we have provided supplementary mathematical analysis to justify its design and demonstrate its rationality.

**Question 2:**Drift Detection Justification: The Gmean-based drift detection mechanism is reasonable and well-motivated for imbalanced data. However: A short comparison with classical drift detectors (e.g., DDM or ADWIN) would strengthen the positioning. A brief discussion on false alarm robustness would be helpful. Even a conceptual comparison (without additional experiments) would improve completeness.

**Response:**Thank you for your insightful comment. We agree that further clarification of the proposed Gmean-based drift detection mechanism—particularly in comparison with classical methods and in terms of false alarm robustness—would improve the completeness of the manuscript.

In the revised manuscript, we have incorporated a conceptual comparison between the proposed method and classical drift detection approaches such as DDM and ADWIN. Specifically, we clarify that DDM relies on monitoring the overall error rate, which may be dominated by majority-class samples and thus fail to reflect performance degradation in minority classes under imbalanced data streams. Similarly, although ADWIN detects distributional changes within adaptive windows, it does not explicitly account for class-wise performance. In contrast, the proposed Gmean-based mechanism evaluates classification performance across both majority and minority classes, making it more suitable for imbalanced scenarios. Furthermore, we have enhanced the discussion on false alarm robustness. In the revised version, we explicitly explain that, by capturing the balance between class-wise performances, Gmean is less sensitive to fluctuations dominated by majority-class predictions. As a result, it can effectively suppress spurious drift alarms caused by transient variations in the overall error rate, thereby improving the robustness of drift detection under class imbalance.

These improvements have been incorporated into the drift detection section of the manuscript (highlighted in the revised version), where the comparison with classical methods and the robustness analysis are now more clearly articulated.

**Question 3:**Statistical Significance: The performance improvements are consistent, but adding a statistical significance test (e.g., Wilcoxon signed-rank test across datasets) would increase the scientific rigor.

**Response:**Thank you for your insightful comment. We agree that incorporating statistical significance analysis can improve the scientific rigor of the manuscript.

In the revised version, we have introduced both the Friedman test and the Wilcoxon signed-rank test to evaluate the statistical significance of the performance improvements. Specifically, the Friedman test is first conducted to examine whether there are overall significant differences among all compared methods. The results show that the p-values for both G-Mean and D(Rec,Spe) are far below 0.05, indicating statistically significant differences across the algorithms. Furthermore, to provide a more detailed pairwise comparison, the Wilcoxon signed-rank test is performed across multiple datasets, comparing EOS-PIELM with each baseline method. The corresponding results are presented in Table 5. The analysis shows that EOS-PIELM achieves statistically significant improvements over most baseline methods. In particular, it demonstrates significant improvements (p < 0.05 or p < 0.01) in both G-Mean and D(Rec,Spe) for the majority of methods, while for WOS-ELM, the improvement is statistically significant in Gmean. In addition, we have included a detailed discussion of these statistical results in the revised manuscript to clearly interpret the significance levels and their implications. These additions enhance the rigor of the experimental evaluation and further support the effectiveness of the proposed EOS-PIELM in handling class imbalance and concept drift in data stream scenarios.

**Question 4:**Computational Complexity Discussion: Since the framework introduces ensemble learning and adaptive updates, it would be beneficial to include:A short time-complexity discussion,A comparison with standard OS-ELM in terms of computational overhead.

**Response:**Thank you for your valuable comment. We agree that a clearer discussion of computational complexity, along with a comparison to the standard OS-ELM, would enhance the completeness of the manuscript.

In the revised manuscript, we have added a dedicated subsection to analyze the time complexity of the proposed EOS-PIELM framework. First, we explicitly present the computational complexity of the standard OS-ELM as a baseline, where the per-instance cost is O(dL + L²). Then, we analyze the complexity of OS-PIELM by considering the additional interference layer, and further extend the analysis to EOS-PIELM, which incorporates an ensemble learning strategy. In particular, we show that EOS-PIELM increases the per-instance computational cost from O(T₀) to O(M × T₁), where M is the number of base classifiers. We also clarify that the additional costs introduced by the voting mechanism and component updates are negligible compared to the main model update operations. Moreover, we emphasize that since M is typically a small constant, the increase in computational complexity is linear and remains computationally efficient in practical applications. Therefore, the proposed method achieves improved performance while maintaining a comparable level of computational overhead to OS-ELM. These revisions have been incorporated into the manuscript to provide a clearer and more rigorous analysis of computational complexity.

**Question 4:** Language and Minor Editorial Improvements:The manuscript is generally understandable but would benefit from: Minor grammatical corrections, Slight refinement of phrasing in the methodology section, Clarification of certain formula transitions. A light professional English editing pass is recommended.

**Response:**We sincerely thank the reviewer for the helpful suggestions. We have carefully revised the manuscript to address the language and editorial issues. In particular, we have made minor grammatical corrections, refined the phrasing in the methodology section, and improved the clarity of several formula transitions to enhance readability. Additionally, a professional English editing pass has been performed to further improve the overall quality of the manuscript.

**Other revisions:** Figure 2 has been revised to improve clarity and readability. Specifically, a legend has been added, and the layout of the modules has been adjusted to enhance the overall visual structure.
