# Supplementary material for: Ensemble learning-based online sequential pre-interference extreme learning for concept drifting and class imbalanced data streams
Source: PLoS One. 2026 Jul 31;21(7):e0353728. doi: 10.1371/journal.pone.0353728 (PMC13426951; doi:10.1371/journal.pone.0353728)
Supplement: Attachment — Submitted filename: Response to Reviewers.doc [file pone.0353728.s003.doc]

**Question 1:**Right now, the introduction talks about concept drift, OS-ELM, and class imbalance, but the review still feels a bit selective and not fully up to date.

**Response:**Thank you for this valuable comment. Following the reviewer’s suggestion, we have revised the Introduction to include more recent related work. In particular, we have added and discussed the study by Li et al. (2024), which demonstrates that conventional machine learning algorithms often exhibit suboptimal performance on highly imbalanced real-world datasets. This work further supports the limitation of relying on a single classifier under severe class imbalance conditions and strengthens the motivation for developing more robust imbalance-aware and ensemble-based learning approaches. The revised manuscript provides a more up-to-date and comprehensive literature review.

**Question 2:**The proposed “pre-interference layer” seems close to nonlinear kernel mapping or random feature transformation, so the authors should clearly say how it is different from kernel ELM, random feature ELM, or other feature-mapping-based online learners.

**Response:**Thank you for this valuable comment. We agree that it is necessary to more clearly distinguish the proposed Pre-Interference Layer from existing feature-mapping-based methods. Following the reviewer’s suggestion, we have revised Section X by adding a detailed comparison with Kernel ELM, random feature-based ELM, and representative online feature-mapping approaches (e.g., OS-KELM). The revised manuscript clarifies that Kernel ELM achieves nonlinear representation by constructing a kernel matrix to model pairwise relationships among training samples, whereas random feature-based methods project samples into a high-dimensional feature space through randomly generated mapping parameters. Furthermore, OS-KELM extends kernel-based mapping to online learning environments; however, its nonlinear representation capability still relies on sample relationship modeling through kernel functions.

In contrast, the proposed Pre-Interference Layer neither constructs a kernel matrix nor employs random feature mapping. Instead, it directly performs nonlinear feature reconstruction on each incoming sample through a set of interference nodes before hidden-layer learning. Therefore, the proposed method focuses on sample-wise feature transformation rather than pairwise sample relationship modeling. This design enhances feature separability while preserving the sequential updating mechanism of OS-ELM, making it more suitable for online data stream learning scenarios.

The corresponding explanations have been incorporated into the revised manuscript to further highlight the motivation behind the proposed Pre-Interference Layer and to clarify its fundamental differences from Kernel ELM, random feature-based methods, and online feature-mapping learners such as OS-KELM.

**Question 3:**The abstract and method description use long sentences with grammar problems.

**Response:**Thank you for this valuable comment. We acknowledge that the abstract and method sections in the original manuscript contained several long and complex sentences that may reduce readability. In the revised version, we have thoroughly revised these parts by shortening overly long sentences, correcting grammatical issues, and restructuring expressions to improve clarity and readability. We believe these revisions have significantly enhanced the overall linguistic quality of the manuscript.

**Question 4:**Algorithm 1 is too vague to reproduce the method.

**Response:**Thank you for this valuable comment. We have revised Algorithm 1 to improve clarity and reproducibility. In the updated version, the complete workflow of EOS-PIELM is explicitly presented, including dynamic weighting, performance evaluation (P0 and P1), Gmean-based drift detection, adaptive parameter update (λ), and classifier update (CF). Compared with the original version, the revised algorithm clearly defines the input–output relationship of each stage, making the overall procedure fully reproducible. Furthermore, to address the concern regarding the Gmean-based drift detection mechanism, we emphasize that it is not a simple combination of existing modules. Instead, the Gmean metric serves as a unified performance indicator to jointly evaluate class imbalance sensitivity and temporal performance variation. The drift decision is directly driven by the comparison between P0 and P1 under this metric, which integrates both historical and current model performance in a consistent framework rather than independently applying multiple existing techniques.

**Question 5:**The explanation around the Gmean-based drift detector could better distinguish real improvement from just adding several known modules together.

**Response:**Thank you for this insightful comment. We agree that the role of the Gmean-based drift detection mechanism should be clarified more explicitly. In the revised manuscript, we have expanded the corresponding discussion to emphasize that the proposed method is not a simple combination of existing modules. Specifically, Gmean is employed as a unified performance indicator to jointly reflect concept drift and class imbalance. The resulting drift information is further used to adaptively adjust the forgetting factor and update ensemble components. Therefore, the proposed mechanism forms a closed-loop adaptation framework that links drift detection, model adaptation, and imbalance-aware learning.

**Question 6:**Since the paper claims to jointly handle concept drift and class imbalance, the authors should more directly connect each experimental result to that claim, rather than only reporting that EOS-PIELM performs better overall.

**Response:**Thank you for this valuable comment. Following the reviewer’s suggestion, we have further improved the experimental analysis section to more explicitly demonstrate the effectiveness of the proposed method in handling concept drift and class imbalance.

Specifically, we have added a more detailed discussion on the practical significance of two evaluation metrics, namely Gmean and D(Rec,Spe). Since Gmean simultaneously considers both minority-class recall and majority-class recognition performance, it provides a comprehensive measure of classification performance under imbalanced data streams. In contrast, D(Rec,Spe) is used to evaluate the balance between the recognition capabilities of different classes. Through this additional discussion, we further illustrate the contribution of the dynamic weighting strategy in alleviating the class imbalance problem. In addition, we further analyze the performance variations before and after concept drift points as shown in Figure 7. The results indicate that EOS-PIELM can recover classification performance more rapidly after the occurrence of concept drift and maintain a more stable Gmean trend. This further verifies that the proposed Gmean-based drift detection mechanism and adaptive forgetting factor effectively enhance the model’s adaptability to concept drift. Therefore, the revised manuscript strengthens the correspondence between the experimental results and the two main objectives of the proposed method, namely class imbalance handling capability and concept drift adaptation capability, thereby more clearly supporting the technical contributions of this study.
