# Supplementary material for: Ensemble learning-based online sequential pre-interference extreme learning for concept drifting and class imbalanced data streams
Source: PLoS One. 2026 Jul 31;21(7):e0353728. doi: 10.1371/journal.pone.0353728 (PMC13426951; doi:10.1371/journal.pone.0353728)
Supplement: Peer review history

# Peer review: Decision Letter 0

**DOI:** 10.1371/journal.pone.0353728.r001

**Authors:** Zeheng Wang

17 Mar 2026

PONE-D-25-66864

Ensemble learning-based online sequential pre-interference extreme learning for concept drifting and class imbalanced data streams

PLOS One

Dear Dr. Wen,

Thank you for submitting your manuscript to PLOS ONE. After careful consideration, we feel that it has merit but does not fully meet PLOS ONE’s publication criteria as it currently stands. Therefore, we invite you to submit a revised version of the manuscript that addresses the points raised during the review process.

Please submit your revised manuscript by May 01 2026 11:59PM. If you will need more time than this to complete your revisions, please reply to this message or contact the journal office at plosone@plos.org. When you're ready to submit your revision, log on to https://www.editorialmanager.com/pone/ and select the 'Submissions Needing Revision' folder to locate your manuscript file.

Please include the following items when submitting your revised manuscript:

- A letter that responds to each point raised by the academic editor and reviewer(s). You should upload this letter as a separate file labeled 'Response to Reviewers'.
- A marked-up copy of your manuscript that highlights changes made to the original version. You should upload this as a separate file labeled 'Revised Manuscript with Track Changes'.
- An unmarked version of your revised paper without tracked changes. You should upload this as a separate file labeled 'Manuscript'.

If you would like to make changes to your financial disclosure, please include your updated statement in your cover letter. Guidelines for resubmitting your figure files are available below the reviewer comments at the end of this letter.

If applicable, we recommend that you deposit your laboratory protocols in protocols.io to enhance the reproducibility of your results. Protocols.io assigns your protocol its own identifier (DOI) so that it can be cited independently in the future. For instructions see: https://journals.plos.org/plosone/s/submission-guidelines#loc-laboratory-protocols. Additionally, PLOS ONE offers an option for publishing peer-reviewed Lab Protocol articles, which describe protocols hosted on protocols.io. Read more information on sharing protocols at https://plos.org/protocols?utm_medium=editorial-email&utm_source=authorletters&utm_campaign=protocols.

We look forward to receiving your revised manuscript.

Kind regards,

Zeheng Wang

Academic Editor

PLOS One

Journal Requirements:

When submitting your revision, we need you to address these additional requirements.

1. Please ensure that your manuscript meets PLOS ONE's style requirements, including those for file naming. The PLOS ONE style templates can be found at

https://journals.plos.org/plosone/s/file?id=wjVg/PLOSOne_formatting_sample_main_body.pdf and https://journals.plos.org/plosone/s/file?id=ba62/PLOSOne_formatting_sample_title_authors_affiliations.pdf

2. Please note that PLOS One has specific guidelines on code sharing for submissions in which author-generated code underpins the findings in the manuscript. In these cases, we expect all author-generated code to be made available without restrictions upon publication of the work. Please review our guidelines at https://journals.plos.org/plosone/s/materials-and-software-sharing#loc-sharing-code and ensure that your code is shared in a way that follows best practice and facilitates reproducibility and reuse.

3. Please provide a complete Data Availability Statement in the submission form, ensuring you include all necessary access information or a reason for why you are unable to make your data freely accessible. If your research concerns only data provided within your submission, please write "All data are in the manuscript and/or supporting information files" as your Data Availability Statement.

4. Thank you for stating the following financial disclosure:

This work is supported by the Fujian Provincial Natural Science Foundation Project (2023J011015, 2022J011179, 2024J01888), Ministry of Education Industry-Academia Collaborative Education Project(231102311285117), Putian Science and Technology Plan Project(2024NJJ009, 2023GJGZ003,

2024

GZ2001PTXY17), Putian University Graduate Research and Innovation Project(yjs2024054), Fujian Province Science and Technology Special Envoy Project(F2022KTP027, F2024KTP086), National Natural Science Foundation of China (grant number 52577115), Putian High end Equipment Industry Technology Research Institute(2023GJGZ002).

Please state what role the funders took in the study. If the funders had no role, please state: "The funders had no role in study design, data collection and analysis, decision to publish, or preparation of the manuscript."

If this statement is not correct you must amend it as needed.

Please include this amended Role of Funder statement in your cover letter; we will change the online submission form on your behalf.

5. Thank you for stating the following in the Acknowledgments Section of your manuscript:

This work is supported by the Fujian Provincial Natural Science Foundation Project (2023J011015, 2022J011179, 2024J01888), Ministry of Education Industry-Academia Collaborative Education Project(231102311285117), Putian Science and Technology Plan Project(2024NJJ009, 2023GJGZ003, 2024 GZ2001PTXY17), Putian University Graduate Research and Innovation Project(yjs2024054), Fujian Province Science and Technology Special Envoy Project(F2022KTP027, F2024KTP086), National Natural Science Foundation of China (grant number 52577115), Putian High end Equipment Industry Technology Research Institute(2023GJGZ002).

We note that you have provided funding information that is not currently declared in your Funding Statement. However, funding information should not appear in the Acknowledgments section or other areas of your manuscript. We will only publish funding information present in the Funding Statement section of the online submission form.

Please remove any funding-related text from the manuscript and let us know how you would like to update your Funding Statement. Currently, your Funding Statement reads as follows:

This work is supported by the Fujian Provincial Natural Science Foundation Project (2023J011015, 2022J011179, 2024J01888), Ministry of Education Industry-Academia Collaborative Education Project(231102311285117), Putian Science and Technology Plan Project(2024NJJ009, 2023GJGZ003,

2024

GZ2001PTXY17), Putian University Graduate Research and Innovation Project(yjs2024054), Fujian Province Science and Technology Special Envoy Project(F2022KTP027, F2024KTP086), National Natural Science Foundation of China (grant number 52577115), Putian High end Equipment Industry Technology Research Institute(2023GJGZ002).

Please include your amended statements within your cover letter; we will change the online submission form on your behalf.

6. Please amend your list of authors on the manuscript to ensure that each author is linked to an affiliation. Authors’ affiliations should reflect the institution where the work was done (if authors moved subsequently, you can also list the new affiliation stating “current affiliation:….” as necessary).

7. Please include a caption for figure 1.

8. We note that Figure(s) 1-4 in your submission contain copyrighted images. All PLOS content is published under the Creative Commons Attribution License (CC BY 4.0), which means that the manuscript, images, and Supporting Information files will be freely available online, and any third party is permitted to access, download, copy, distribute, and use these materials in any way, even commercially, with proper attribution. For more information, see our copyright guidelines: http://journals.plos.org/plosone/s/licenses-and-copyright.

We require you to either (1) present written permission from the copyright holder to publish these figures specifically under the CC BY 4.0 license, or (2) remove the figures from your submission:

a. You may seek permission from the original copyright holder of Figure(s) 1-4 to publish the content specifically under the CC BY 4.0 license.

We recommend that you contact the original copyright holder with the Content Permission Form (http://journals.plos.org/plosone/s/file?id=7c09/content-permission-form.pdf) and the following text:

“I request permission for the open-access journal PLOS ONE to publish XXX under the Creative Commons Attribution License (CCAL) CC BY 4.0 (http://creativecommons.org/licenses/by/4.0/). Please be aware that this license allows unrestricted use and distribution, even commercially, by third parties. Please reply and provide explicit written permission to publish XXX under a CC BY license and complete the attached form.”

Please upload the completed Content Permission Form or other proof of granted permissions as an "Other" file with your submission.

In the figure caption of the copyrighted figure, please include the following text: “Reprinted from [ref] under a CC BY license, with permission from [name of publisher], original copyright [original copyright year].”

b. If you are unable to obtain permission from the original copyright holder to publish these figures under the CC BY 4.0 license or if the copyright holder’s requirements are incompatible with the CC BY 4.0 license, please either i) remove the figure or ii) supply a replacement figure that complies with the CC BY 4.0 license. Please check copyright information on all replacement figures and update the figure caption with source information. If applicable, please specify in the figure caption text when a figure is similar but not identical to the original image and is therefore for illustrative purposes only.

9. If the reviewer comments include a recommendation to cite specific previously published works, please review and evaluate these publications to determine whether they are relevant and should be cited. There is no requirement to cite these works unless the editor has indicated otherwise.

[Note: HTML markup is below. Please do not edit.]

Reviewers' comments:

Reviewer's Responses to Questions

**Comments to the Author**

1. Is the manuscript technically sound, and do the data support the conclusions?

The manuscript must describe a technically sound piece of scientific research with data that supports the conclusions. Experiments must have been conducted rigorously, with appropriate controls, replication, and sample sizes. The conclusions must be drawn appropriately based on the data presented.

Reviewer #1: Yes

Reviewer #2: Yes

Reviewer #3: Yes

**********

2. Has the statistical analysis been performed appropriately and rigorously?

Reviewer #1: Yes

Reviewer #2: Yes

Reviewer #3: Yes

**********

3. Have the authors made all data underlying the findings in their manuscript fully available?

The PLOS Data policy requires authors to make all data underlying the findings described in their manuscript fully available without restriction, with rare exception (please refer to the Data Availability Statement in the manuscript PDF file). The data should be provided as part of the manuscript or its supporting information, or deposited to a public repository. For example, in addition to summary statistics, the data points behind means, medians and variance measures should be available. If there are restrictions on publicly sharing data—e.g. participant privacy or use of data from a third party—those must be specified.

Reviewer #1: Yes

Reviewer #2: Yes

Reviewer #3: Yes

**********

4. Is the manuscript presented in an intelligible fashion and written in standard English?

PLOS ONE does not copyedit accepted manuscripts, so the language in submitted articles must be clear, correct, and unambiguous. Any typographical or grammatical errors should be corrected at revision, so please note any specific errors here.

Reviewer #1: Yes

Reviewer #2: Yes

Reviewer #3: Yes

**********

5. Review Comments to the Author

Please use the space provided to explain your answers to the questions above. You may also include additional comments for the author, including concerns about dual publication, research ethics, or publication ethics. (Please upload your review as an attachment if it exceeds 20,000 characters)

Reviewer #1: Revised Review Comments�

This paper proposes an online learning algorithm called OS-PIELM, which enhances the discriminative power of data stream features by introducing an interference layer between the input and hidden layers of the traditional OS-ELM for nonlinear kernel mapping. The method also integrates an adaptive concept drift detection mechanism based on G-means and a dynamic weighting strategy, enabling it to simultaneously handle class imbalance and concept drift problems. Overall, the methodology is highly innovative, but it still has the following shortcomings:

1. What is the relationship between the proposed concept drift and class imbalance? Is it the result of problem A + problem B, or an intersection of problem A and problem B? This needs to be explained in detail.

2. Supplement the description of Fig. 3 (Problem: The descriptions of different figures are completely identical).

3. The ordinate of Fig. 6 does not match the description.

4. The punctuation in "comparison among and other methods" is incorrect.

5. Supplement the description of the comparative model.

6. Add an explanation of "virtual drift," etc.

7. Supplement the width of the drift.

8. The authors need to supplement the discussion of the broader field of stream learning, particularly regarding the current state of research in online learning, and point out the connections and differences between the proposed method and related works to better illustrate the work presented in this paper. This includes, but is not limited to, the following outstanding works:

Online Learning from Mix-typed, Drifted, and Incomplete Streaming Features (TKDD)

Online Semi-supervised Learning with Mix-Typed Streaming Features (AAAI)

Online Learning for Data Streams With Incomplete Features and Labels (TKDE)

Reviewer #2: Reviewer Comments

1. Inconsistent Terminology Usage

The key terms pre-interference layer and interference layer are used interchangeably throughout the manuscript to refer to the same structural component added to the OS-ELM model. Additionally, the concept of class imbalance is described with two inconsistent expressions: class imbalanced and category imbalance. Such terminological inconsistency violates the basic norms of academic writing and may cause confusion for readers in understanding the core design and research object of the study.

2. Grammatical Errors and Inadequate Proofreading

The manuscript contains multiple grammatical errors and spelling mistakes, which reflect a lack of careful proofreading. For example, the conjunction redundancy in the sentence "Although the interference layer... but the class labels..." (the simultaneous use of Although and but violates English grammatical rules); spelling errors such as Performce (correct: Performance) and ndr rves (correct: curves) in the text and figure captions. These issues significantly reduce the readability and formality of the manuscript.

3. Insufficient Interpretation of Key Figures

Key experimental figures (Figure 5, Figure 6, Figure 7) are only briefly referenced with a general conclusion in the text, without detailed data-driven interpretation and analysis. The manuscript fails to elaborate on the specific trends, critical inflection points, and statistical characteristics of the experimental results presented in these figures, nor does it link the figure data to the core research hypotheses and algorithm performance conclusions of the study. This makes the experimental results lack sufficient empirical support and weakens the persuasiveness of the research findings.

4. Superficial Review of Existing Literature and Unclear Research Gaps

The review of existing research merely lists the improved methods of OS-ELM for data stream learning in a descriptive manner, without systematically sorting out and summarizing the core research challenges in the field of class-imbalanced data stream learning with concept drift (e.g., poor coupling of multiple functional modules, inadequate handling of multi-class classification scenarios, weak noise resistance of algorithms). More importantly, the manuscript does not explicitly establish the corresponding relationship between the identified research gaps and the innovative points of the proposed EOS-PIELM algorithm, which makes it difficult for readers to recognize the theoretical and practical contributions of this study relative to the existing literature.

Reviewer #3: 1. Overall Evaluation

This manuscript proposes an online ensemble learning framework (EOS-PIELM) designed to simultaneously address two major challenges in data stream learning: concept drift and class imbalance. The authors extend OS-ELM by introducing:

A pre-interference nonlinear mapping layer (OS-PIELM),

A Gmean-based concept drift detection mechanism with an adaptive forgetting factor,

A dynamic class-weighting strategy,

An online ensemble framework with performance-based voting.

The topic is timely and practically relevant, especially for real-world streaming scenarios where distribution shifts and imbalance frequently co-occur. The manuscript is generally well-structured, and the experimental results demonstrate consistent performance improvements across synthetic and real-world datasets.

Overall, the work presents a meaningful incremental advancement in online ELM-based stream learning and is suitable for publication after addressing several clarifications and improvements.

2. Strengths of the Manuscript

2.1 Practical Relevance

The simultaneous treatment of concept drift and class imbalance reflects realistic industrial and real-time data environments (e.g., weather forecasting and electricity pricing). The proposed framework directly targets these combined challenges.

2.2 Systematic Framework Design

The algorithm integrates multiple components coherently:

Nonlinear feature enhancement via the pre-interference layer,

Drift detection grounded in Gmean performance,

Adaptive forgetting factor adjustment,

Cost-sensitive dynamic reweighting,

Ensemble voting with classifier-specific correction factors.

The overall architecture is logically constructed and technically sound.

2.3 Empirical Performance

Experimental results show:

Consistently higher Gmean values compared to OS-ELM variants,

Strong robustness under varying imbalance ratios,

Stable behavior on both abrupt and gradual drift,

Improved performance on real-world datasets (Weather and Elec).

The inclusion of multiple evaluation metrics (Accuracy, Recall, Specificity, Gmean, D(Rec, Spe)) strengthens the empirical analysis.

3. Suggestions for Improvement

While the manuscript is promising, several points could be clarified or strengthened to improve readability and rigor.

3.1 Clarify the Role of the Pre-Interference Layer

The pre-interference layer is a key contribution. However, its theoretical motivation could be elaborated further:

Is ϕ(x) fixed or randomly initialized?

How does this differ from standard kernel ELM or random feature mapping?

Does it increase representational capacity theoretically?

A brief discussion on its theoretical interpretation would enhance clarity.

3.2 Drift Detection Justification

The Gmean-based drift detection mechanism is reasonable and well-motivated for imbalanced data. However:

A short comparison with classical drift detectors (e.g., DDM or ADWIN) would strengthen the positioning.

A brief discussion on false alarm robustness would be helpful.

Even a conceptual comparison (without additional experiments) would improve completeness.

3.3 Statistical Significance

The performance improvements are consistent, but adding a statistical significance test (e.g., Wilcoxon signed-rank test across datasets) would increase the scientific rigor.

3.4 Computational Complexity Discussion

Since the framework introduces ensemble learning and adaptive updates, it would be beneficial to include:

A short time-complexity discussion,

A comparison with standard OS-ELM in terms of computational overhead.

Even a qualitative complexity analysis would suffice.

3.5 Language and Minor Editorial Improvements

The manuscript is generally understandable but would benefit from:

Minor grammatical corrections,

Slight refinement of phrasing in the methodology section,

Clarification of certain formula transitions.

A light professional English editing pass is recommended.

4. Conclusion

This work presents a well-designed and practically motivated extension of OS-ELM for handling both concept drift and class imbalance in data streams. The integration of nonlinear feature mapping, adaptive forgetting, and dynamic class reweighting within an ensemble framework is coherent and experimentally validated.

The manuscript demonstrates clear performance advantages over several baseline methods and provides sufficient empirical support.

With minor clarifications and modest revisions to improve theoretical explanation and presentation quality, the paper would be suitable for publication.

**********

6. PLOS authors have the option to publish the peer review history of their article (what does this mean?). If published, this will include your full peer review and any attached files.

If you choose “no”, your identity will remain anonymous but your review may still be made public.

**Do you want your identity to be public for this peer review?**  For information about this choice, including consent withdrawal, please see our Privacy Policy.

Reviewer #1: No

Reviewer #2: No

Reviewer #3: No

**********

[NOTE: If reviewer comments were submitted as an attachment file, they will be attached to this email and accessible via the submission site. Please log into your account, locate the manuscript record, and check for the action link "View Attachments". If this link does not appear, there are no attachment files.]

To ensure your figures meet our technical requirements, please review our figure guidelines: https://journals.plos.org/plosone/s/figures

You may also use PLOS’s free figure tool, NAAS, to help you prepare publication quality figures: https://journals.plos.org/plosone/s/figures#loc-tools-for-figure-preparation.

NAAS will assess whether your figures meet our technical requirements by comparing each figure against our figure specifications.

# Author comment: Author response to Decision Letter 1

**DOI:** 10.1371/journal.pone.0353728.r002

28 Apr 2026

Dear Dr. Wang,

Thank you for your email and for outlining the additional requirements for our manuscript. We have carefully addressed all the points raised and revised our submission accordingly. Our responses are detailed below:

Question 1�Please ensure that your manuscript meets PLOS ONE's style requirements, including those for file naming. The PLOS ONE style templates can be found at

https://journals.plos.org/plosone/s/file?id=wjVg/PLOSOne_formatting_sample_main_body.pdf and https://journals.plos.org/plosone/s/file?id=ba62/PLOSOne_formatting_sample_title_authors_affiliations.pdf

Response:We have revised the manuscript to ensure full compliance with PLOS ONE formatting guidelines, including file structure, organization, and naming conventions. The manuscript has been updated using the provided templates to meet all journal requirements.

Question 2:Please note that PLOS One has specific guidelines on code sharing for submissions in which author-generated code underpins the findings in the manuscript. In these cases, we expect all author-generated code to be made available without restrictions upon publication of the work. Please review our guidelines at https://journals.plos.org/plosone/s/materials-and-software-sharing#loc-sharing-code and ensure that your code is shared in a way that follows best practice and facilitates reproducibility and reuse.

Response:We thank the editor for this comment. In accordance with PLOS ONE’s code sharing guidelines, we have made the source code used in this study publicly available without restriction. The code is accessible at the following repository:

https://github.com/cutainrob/EOS-PIELM

This repository provides the implementation of the proposed methods and is intended to facilitate transparency, reproducibility, and reuse of our work.

Question 3:Please provide a complete Data Availability Statement in the submission form, ensuring you include all necessary access information or a reason for why you are unable to make your data freely accessible. If your research concerns only data provided within your submission, please write "All data are in the manuscript and/or supporting information files" as your Data Availability Statement.

Response:The data used in this study consist of artificially generated datasets and publicly available datasets from the UCI Machine Learning Repository. The specific sources of all datasets have been cited in the manuscript. Accordingly, we have included the following Data Availability Statement in the revised manuscript:

“All data underlying the findings in this study are fully available without restriction. The artificial datasets were generated according to the procedures described in the manuscript. The public datasets were obtained from the UCI Machine Learning Repository, and their specific sources are cited in the main text.”

Question 4:Thank you for stating the following financial disclosure:

This work is supported by the Fujian Provincial Natural Science Foundation Project (2023J011015, 2022J011179, 2024J01888), Ministry of Education Industry-Academia Collaborative Education Project(231102311285117), Putian Science and Technology Plan Project(2024NJJ009, 2023GJGZ003,2024GZ2001PTXY17), Putian University Graduate Research and Innovation Project(yjs2024054), Fujian Province Science and Technology Special Envoy Project(F2022KTP027, F2024KTP086), National Natural Science Foundation of China (grant number 52577115), Putian High end Equipment Industry Technology Research Institute(2023GJGZ002). Please state what role the funders took in the study. If the funders had no role, please state: "The funders had no role in study design, data collection and analysis, decision to publish, or preparation of the manuscript."

Response:We thank the editor for this comment. We have clarified the role of the funders in both the manuscript and the cover letter. The statement is as follows:

“The funders had no role in study design, data collection and analysis, decision to publish, or preparation of the manuscript.”

Question 5:We note that you have provided funding information that is not currently declared in your Funding Statement. However, funding information should not appear in the Acknowledgments section or other areas of your manuscript. We will only publish funding information present in the Funding Statement section of the online submission form.

Response:We thank the editor for this clarification. We have carefully revised the manuscript and removed all funding information from the Acknowledgments section and any other parts of the main text. The funding information is now provided exclusively in the Funding Statement section of the online submission form, in full compliance with PLOS ONE requirements.

Question 6:Please amend your list of authors on the manuscript to ensure that each author is linked to an affiliation. Authors’ affiliations should reflect the institution where the work was done (if authors moved subsequently, you can also list the new affiliation stating “current affiliation:….” as necessary).

Response:We thank the editor for this comment. We have carefully reviewed the author list and affiliations in the manuscript. Each author is correctly linked to their respective affiliation, and the affiliations accurately reflect the institutions where the work was conducted. No inconsistencies were identified, and the formatting has been checked to ensure compliance with the journal’s requirements.

Question 7:Please include a caption for figure 1.

Response:We thank the editor for this comment. We have added a caption for Figure 1 in the revised manuscript. The figure caption has been included in the main text below the figure, in accordance with the journal’s formatting requirements.

Question 8:We note that Figure(s) 1-4 in your submission contain copyrighted images. All PLOS content is published under the Creative Commons Attribution License (CC BY 4.0), which means that the manuscript, images, and Supporting Information files will be freely available online, and any third party is permitted to access, download, copy, distribute, and use these materials in any way, even commercially, with proper attribution. For more information, see our copyright guidelines: http://journals.plos.org/plosone/s/licenses-and-copyright.

Response:We thank the editor for this comment. We confirm that Figures 1–4 in the manuscript are original figures created by the authors. They do not contain any copyrighted material from third-party sources. Therefore, they are fully compliant with the Creative Commons Attribution License (CC BY 4.0) requirements of PLOS ONE.

Reviewer #1:

Question 1�What is the relationship between the proposed concept drift and class imbalance? Is it the result of problem A + problem B, or an intersection of problem A and problem B? This needs to be explained in detail.

Response:Thank you for this valuable comment. We agree that the relationship between concept drift and class imbalance was not sufficiently clarified in the original manuscript. In the revised version, we have improved the corresponding description to explicitly explain their relationship.

First, we introduce a practical bearing fault diagnosis scenario to illustrate that both concept drift and class imbalance commonly occur in data streams. Specifically, variations in operating conditions lead to dynamically changing data distributions, resulting in concept drift, while the scarcity of fault samples in early stages leads to class imbalance. More importantly, we clarify that these two issues are not independent but inherently coupled. Concept drift can alter class prior probabilities or class-conditional distributions, thereby causing the degree of class imbalance to evolve dynamically over time. Conversely, class imbalance further increases the difficulty of learning in drifting data streams, particularly by degrading the recognition performance of minority classes, and may adversely affect online learning models, including concept drift detection mechanisms. Accordingly, we emphasize that the problem addressed in this work is not a simple combination of two independent challenges, but a coupled learning problem under both concept drift and class imbalance. The revised manuscript has been updated to reflect this clarification.

Question 2:Supplement the description of Fig. 3 (Problem: The descriptions of different figures are completely identical).

Response:We fully agree with your suggestion. To enhance the interpretability of Figure 3, we have added the following explanation in the first paragraph of the OS-PIELM subsection:

“As shown in Fig. 3, the upper subfigure illustrates a pre-interference layer with two nodes, while the lower subfigure illustrates a pre-interference layer with three nodes. The orange rectangles and green circles denote two different classes of samples. After nonlinear kernel mapping of the original samples, the samples become more separable in the transformed feature space, indicating that the proposed interference layer enhances the discriminative representation of the data.” This revision has been incorporated into the revised manuscript.

Question 3:The ordinate of Fig. 6 does not match the description.

Response:We appreciate your careful review and constructive feedback. Upon verification, we found that there was a labeling error in the vertical axis of Figure 6. We have replaced the original figure, corrected the vertical axis label by changing “G-Mean” to “Gmean,” and revised the figure caption accordingly: Fig 6. Gmean of EOS-PIELM under different under different sizes of data chunks for synthetic and real-time data streams

Question 4�The punctuation in "comparison among and other methods" is incorrect.

Response:Thank you for your careful review. We agree that the expression “comparison among and other methods” in the title is grammatically incorrect. It has been revised to “Comparison with other methods”to ensure clarity and correctness. The revision has been incorporated into the manuscript.

Question 5�Supplement the description of the comparative model

Response:Thank you for the valuable comment. We agree that a clearer description of the comparative models can help readers better understand their characteristics and the rationale for selecting them as baselines. In the revised manuscript, we have added brief descriptions of all comparative methods, including VWOS-ELM, WOS-ELM, FROS-ELM, OS-ELM, LPP, and SRP, to clarify their main mechanisms and application scenarios. The corresponding descriptions have been added in Comparison methods.

Question 6�Add an explanation of "virtual drift," etc.

Response:We sincerely thank the reviewer for the valuable suggestion. We agree that providing a clearer explanation of “virtual drift” can improve readers’ understanding of this concept. In the revised manuscript, we have added a more detailed description of virtual drift and further clarified the distinction between virtual drift and real concept drift.

Question 7:Supplement the width of the drift.

Response:We sincerely thank the reviewer for the valuable suggestion. We agree that explicitly specifying the drift width in the data stream experimental settings helps to more clearly characterize changes in data distribution. In the revised manuscript, we have added a detailed description of the drift width in the Sine_IR data generator. Since this dataset simulates abrupt concept drift by directly flipping the decision boundary, the drift width is set to 1, indicating that the change in data distribution occurs instantaneously. For the Sea_IR_T data generator, the drift width is set to 1 for abrupt concept drift, while it is set to 1000 for gradual drift, indicating that the data distribution transitions progressively over a certain interval. The corresponding explanations have been added to the “Datasets” subsection under “Experimental Results and Discussion” in the revised manuscript.

Question 8:The authors need to supplement the discussion of the broader field of stream learning, particularly regarding the current state of research in online learning, and point out the connections and differences between the proposed method and related works to better illustrate the work presented in this paper.

Response:Thank you for your valuable comment. We agree that a clearer discussion of the broader field of stream learning, as well as the connections and differences between related works and the proposed method, would improve the clarity and completeness of the manuscript.

In the revised manuscript, we have expanded the introduction to include a more comprehensive review of recent advances in online learning for data streams. Specifically, we discuss representative methods addressing different challenges, including incomplete or uncertain data (e.g., OLIFL), concept drift adaptation, and semi-supervised learning. For each category, we not only describe the main ideas and advantages but also explicitly analyze their limitations. Furthermore, we have clarified the connections and differences between these existing approaches and the proposed method. The revised discussion highlights that most existing methods focus on a single aspect of data stream learning and lack the ability to simultaneously address multiple interacting challenges, particularly the joint presence of concept drift and class imbalance. Based on this analysis, we explicitly identify the research gap and motivate the proposed EOS-PIELM framework, which integrates concept drift detection, adaptive forgetting mechanisms, and class imbalance handling into a unified online learning framework.

These revisions have been incorporated into the introduction section of the manuscript to improve its logical coherence and to better position the contributions of this work within the broader research landscape.

Reviewer #2

Question 1�The key terms pre-interference layer and interference layer are used interchangeably throughout the manuscript to refer to the same structural component added to the OS-ELM model. Additionally, the concept of class imbalance is described with two inconsistent expressions: class imbalanced and category imbalance. Such terminological inconsistency violates the basic norms of academic writing and may cause confusion for readers in understanding the core design and research object of the study.

Response:Thank you for the careful observation. We agree that consistent terminology is essential for clarity in academic writing. In the revised manuscript, we have unified the terminology throughout the paper. Specifically, the term “pre-interference layer” is consistently used to refer to the structural component inserted between the input and hidden layers of the OS-ELM model. In addition, the expression “class imbalance” is used uniformly to describe the imbalance problem in data streams. All inconsistent terms have been corrected accordingly in the revised manuscript.

Question 2�Grammatical Errors and Inadequate Proofreading�The manuscript contains multiple grammatical errors and spelling mistakes, which reflect a lack of careful proofreading. For example, the conjunction redundancy in the sentence "Although the interference layer... but the class labels..." (the simultaneous use of Although and but violates English grammatical rules); spelling errors such as Performce (correct: Performance) and ndr rves (correct: curves) in the text and figure captions. These issues significantly reduce the readability and formality of the manuscript.

Response:Thank you for the careful review and for pointing out these language issues. We agree that grammatical accuracy and consistent proofreading are essential for maintaining the clarity and readability of the manuscript. In the revised version, we have carefully proofread the entire manuscript and corrected the grammatical and spelling errors identified by the reviewer. Specifically, redundant conjunctions such as the simultaneous use of “Although” and “but” have been corrected, and spelling errors in both the main text and figure captions (e.g., “Performancece” and “ndr rves”) have been fixed. In addition, the manuscript has been thoroughly checked to ensure linguistic accuracy and consistency throughout the paper.

Attachment Submitted filename: Response_PONE-D-25-66864.docx

# Peer review: Decision Letter 1

**DOI:** 10.1371/journal.pone.0353728.r003

**Authors:** Zeheng Wang

29 May 2026

PONE-D-25-66864R1

Ensemble learning-based online sequential pre-interference extreme learning for concept drifting and class imbalanced data streams

PLOS One

Dear Dr. Wen,

Thank you for submitting your manuscript to PLOS ONE. After careful consideration, we feel that it has merit but does not fully meet PLOS ONE’s publication criteria as it currently stands. Therefore, we invite you to submit a revised version of the manuscript that addresses the points raised during the review process.

Please submit your revised manuscript by Jul 13 2026 11:59PM. If you will need more time than this to complete your revisions, please reply to this message or contact the journal office at plosone@plos.org. When you're ready to submit your revision, log on to https://www.editorialmanager.com/pone/ and select the 'Submissions Needing Revision' folder to locate your manuscript file.

Please include the following items when submitting your revised manuscript:

- A letter that responds to each point raised by the academic editor and reviewer(s). You should upload this letter as a separate file labeled 'Response to Reviewers'.
- A marked-up copy of your manuscript that highlights changes made to the original version. You should upload this as a separate file labeled 'Revised Manuscript with Track Changes'.
- An unmarked version of your revised paper without tracked changes. You should upload this as a separate file labeled 'Manuscript'.

If you would like to make changes to your financial disclosure, please include your updated statement in your cover letter. Guidelines for resubmitting your figure files are available below the reviewer comments at the end of this letter.

If applicable, we recommend that you deposit your laboratory protocols in protocols.io to enhance the reproducibility of your results. Protocols.io assigns your protocol its own identifier (DOI) so that it can be cited independently in the future. For instructions see: https://journals.plos.org/plosone/s/submission-guidelines#loc-laboratory-protocols. Additionally, PLOS ONE offers an option for publishing peer-reviewed Lab Protocol articles, which describe protocols hosted on protocols.io. Read more information on sharing protocols at https://plos.org/protocols?utm_medium=editorial-email&utm_source=authorletters&utm_campaign=protocols.

As the corresponding author, your ORCID iD is verified in the submission system and will appear in the published article. PLOS supports the use of ORCID, and we encourage all coauthors to register for an ORCID iD and use it as well. Please encourage your coauthors to verify their ORCID iD within the submission system before final acceptance, as unverified ORCID iDs will not appear in the published article. *Only*  the individual author can complete the verification step; PLOS staff *cannot*  verify ORCID iDs on behalf of authors.

We look forward to receiving your revised manuscript.

Kind regards,

Zeheng Wang

Academic Editor

PLOS One

Journal Requirements:

1. If the reviewer comments include a recommendation to cite specific previously published works, please review and evaluate these publications to determine whether they are relevant and should be cited. There is no requirement to cite these works unless the editor has indicated otherwise.

2. Please review your reference list to ensure that it is complete and correct. If you have cited papers that have been retracted, please include the rationale for doing so in the manuscript text, or remove these references and replace them with relevant current references. Any changes to the reference list should be mentioned in the rebuttal letter that accompanies your revised manuscript. If you need to cite a retracted article, indicate the article’s retracted status in the References list and also include a citation and full reference for the retraction notice.

[Note: HTML markup is below. Please do not edit.]

Reviewers' comments:

Reviewer's Responses to Questions

**Comments to the Author**

1. If the authors have adequately addressed your comments raised in a previous round of review and you feel that this manuscript is now acceptable for publication, you may indicate that here to bypass the “Comments to the Author” section, enter your conflict of interest statement in the “Confidential to Editor” section, and submit your "Accept" recommendation.

Reviewer #1: All comments have been addressed

Reviewer #3: All comments have been addressed

Reviewer #4: (No Response)

**********

2. Is the manuscript technically sound, and do the data support the conclusions?

The manuscript must describe a technically sound piece of scientific research with data that supports the conclusions. Experiments must have been conducted rigorously, with appropriate controls, replication, and sample sizes. The conclusions must be drawn appropriately based on the data presented.

Reviewer #1: (No Response)

Reviewer #3: Yes

Reviewer #4: Yes

**********

3. Has the statistical analysis been performed appropriately and rigorously?

Reviewer #1: (No Response)

Reviewer #3: Yes

Reviewer #4: Yes

**********

4. Have the authors made all data underlying the findings in their manuscript fully available?

The PLOS Data policy requires authors to make all data underlying the findings described in their manuscript fully available without restriction, with rare exception (please refer to the Data Availability Statement in the manuscript PDF file). The data should be provided as part of the manuscript or its supporting information, or deposited to a public repository. For example, in addition to summary statistics, the data points behind means, medians and variance measures should be available. If there are restrictions on publicly sharing data—e.g. participant privacy or use of data from a third party—those must be specified.

Reviewer #1: (No Response)

Reviewer #3: Yes

Reviewer #4: Yes

**********

5. Is the manuscript presented in an intelligible fashion and written in standard English?

PLOS ONE does not copyedit accepted manuscripts, so the language in submitted articles must be clear, correct, and unambiguous. Any typographical or grammatical errors should be corrected at revision, so please note any specific errors here.

Reviewer #1: (No Response)

Reviewer #3: Yes

Reviewer #4: Yes

**********

6. Review Comments to the Author

Please use the space provided to explain your answers to the questions above. You may also include additional comments for the author, including concerns about dual publication, research ethics, or publication ethics. (Please upload your review as an attachment if it exceeds 20,000 characters)

Reviewer #1: (No Response)

Reviewer #3: (No Response)

Reviewer #4: The paper is interesting and the topic is relevant, but I still feel the manuscript needs a stronger positioning in the current literature. Right now, the introduction talks about concept drift, OS-ELM, and class imbalance, but the review still feels a bit selective and not fully up to date. In particular, the authors should cite and discuss recent work such as Li et al. (2024), “Suboptimal capability of individual machine learning algorithms in modeling small-scale imbalanced clinical data of local hospital,” PLOS ONE 19(2): e0298328. That paper is not about data streams directly, but it is highly relevant because it shows that standard individual ML models can perform poorly on small-scale, highly imbalanced real-world data, which supports the motivation for more robust imbalance-aware and ensemble-based methods. Ignoring this kind of recent evidence makes the paper’s motivation look less complete.

I also think the technical novelty needs to be explained more carefully. The proposed “pre-interference layer” seems close to nonlinear kernel mapping or random feature transformation, so the authors should clearly say how it is different from kernel ELM, random feature ELM, or other feature-mapping-based online learners. Some parts of the manuscript are still not very clear: for example, the abstract and method description use long sentences with grammar problems, Algorithm 1 is too vague to reproduce the method, and the explanation around the Gmean-based drift detector could better distinguish real improvement from just adding several known modules together. Since the paper claims to jointly handle concept drift and class imbalance, the authors should more directly connect each experimental result to that claim, rather than only reporting that EOS-PIELM performs better overall.

**********

7. PLOS authors have the option to publish the peer review history of their article (what does this mean?). If published, this will include your full peer review and any attached files.

If you choose “no”, your identity will remain anonymous but your review may still be made public.

**Do you want your identity to be public for this peer review?**  For information about this choice, including consent withdrawal, please see our Privacy Policy.

Reviewer #1: No

Reviewer #3: No

Reviewer #4: No

**********

[NOTE: If reviewer comments were submitted as an attachment file, they will be attached to this email and accessible via the submission site. Please log into your account, locate the manuscript record, and check for the action link "View Attachments". If this link does not appear, there are no attachment files.]

To ensure your figures meet our technical requirements, please review our figure guidelines: https://journals.plos.org/plosone/s/figures

You may also use PLOS’s free figure tool, NAAS, to help you prepare publication quality figures: https://journals.plos.org/plosone/s/figures#loc-tools-for-figure-preparation.

NAAS will assess whether your figures meet our technical requirements by comparing each figure against our figure specifications.

# Author comment: Author response to Decision Letter 2

**DOI:** 10.1371/journal.pone.0353728.r004

24 Jun 2026

Question 1:Right now, the introduction talks about concept drift, OS-ELM, and class imbalance, but the review still feels a bit selective and not fully up to date.

Response:Thank you for this valuable comment. Following the reviewer’s suggestion, we have revised the Introduction to include more recent related work. In particular, we have added and discussed the study by Li et al. (2024), which demonstrates that conventional machine learning algorithms often exhibit suboptimal performance on highly imbalanced real-world datasets. This work further supports the limitation of relying on a single classifier under severe class imbalance conditions and strengthens the motivation for developing more robust imbalance-aware and ensemble-based learning approaches. The revised manuscript provides a more up-to-date and comprehensive literature review.

Question 2:The proposed “pre-interference layer” seems close to nonlinear kernel mapping or random feature transformation, so the authors should clearly say how it is different from kernel ELM, random feature ELM, or other feature-mapping-based online learners.

Response:Thank you for this valuable comment. We agree that it is necessary to more clearly distinguish the proposed Pre-Interference Layer from existing feature-mapping-based methods. Following the reviewer’s suggestion, we have revised Section X by adding a detailed comparison with Kernel ELM, random feature-based ELM, and representative online feature-mapping approaches (e.g., OS-KELM). The revised manuscript clarifies that Kernel ELM achieves nonlinear representation by constructing a kernel matrix to model pairwise relationships among training samples, whereas random feature-based methods project samples into a high-dimensional feature space through randomly generated mapping parameters. Furthermore, OS-KELM extends kernel-based mapping to online learning environments; however, its nonlinear representation capability still relies on sample relationship modeling through kernel functions.

In contrast, the proposed Pre-Interference Layer neither constructs a kernel matrix nor employs random feature mapping. Instead, it directly performs nonlinear feature reconstruction on each incoming sample through a set of interference nodes before hidden-layer learning. Therefore, the proposed method focuses on sample-wise feature transformation rather than pairwise sample relationship modeling. This design enhances feature separability while preserving the sequential updating mechanism of OS-ELM, making it more suitable for online data stream learning scenarios.

The corresponding explanations have been incorporated into the revised manuscript to further highlight the motivation behind the proposed Pre-Interference Layer and to clarify its fundamental differences from Kernel ELM, random feature-based methods, and online feature-mapping learners such as OS-KELM.

Question 3:The abstract and method description use long sentences with grammar problems.

Response:Thank you for this valuable comment. We acknowledge that the abstract and method sections in the original manuscript contained several long and complex sentences that may reduce readability. In the revised version, we have thoroughly revised these parts by shortening overly long sentences, correcting grammatical issues, and restructuring expressions to improve clarity and readability. We believe these revisions have significantly enhanced the overall linguistic quality of the manuscript.

Question 4:Algorithm 1 is too vague to reproduce the method.

Response:Thank you for this valuable comment. We have revised Algorithm 1 to improve clarity and reproducibility. In the updated version, the complete workflow of EOS-PIELM is explicitly presented, including dynamic weighting, performance evaluation (P0 and P1), Gmean-based drift detection, adaptive parameter update (λ), and classifier update (CF). Compared with the original version, the revised algorithm clearly defines the input–output relationship of each stage, making the overall procedure fully reproducible. Furthermore, to address the concern regarding the Gmean-based drift detection mechanism, we emphasize that it is not a simple combination of existing modules. Instead, the Gmean metric serves as a unified performance indicator to jointly evaluate class imbalance sensitivity and temporal performance variation. The drift decision is directly driven by the comparison between P0 and P1 under this metric, which integrates both historical and current model performance in a consistent framework rather than independently applying multiple existing techniques.

Question 5:The explanation around the Gmean-based drift detector could better distinguish real improvement from just adding several known modules together.

Response:Thank you for this insightful comment. We agree that the role of the Gmean-based drift detection mechanism should be clarified more explicitly. In the revised manuscript, we have expanded the corresponding discussion to emphasize that the proposed method is not a simple combination of existing modules. Specifically, Gmean is employed as a unified performance indicator to jointly reflect concept drift and class imbalance. The resulting drift information is further used to adaptively adjust the forgetting factor and update ensemble components. Therefore, the proposed mechanism forms a closed-loop adaptation framework that links drift detection, model adaptation, and imbalance-aware learning.

Question 6:Since the paper claims to jointly handle concept drift and class imbalance, the authors should more directly connect each experimental result to that claim, rather than only reporting that EOS-PIELM performs better overall.

Response:Thank you for this valuable comment. Following the reviewer’s suggestion, we have further improved the experimental analysis section to more explicitly demonstrate the effectiveness of the proposed method in handling concept drift and class imbalance.

Specifically, we have added a more detailed discussion on the practical significance of two evaluation metrics, namely Gmean and D(Rec,Spe). Since Gmean simultaneously considers both minority-class recall and majority-class recognition performance, it provides a comprehensive measure of classification performance under imbalanced data streams. In contrast, D(Rec,Spe) is used to evaluate the balance between the recognition capabilities of different classes. Through this additional discussion, we further illustrate the contribution of the dynamic weighting strategy in alleviating the class imbalance problem. In addition, we further analyze the performance variations before and after concept drift points as shown in Figure 7. The results indicate that EOS-PIELM can recover classification performance more rapidly after the occurrence of concept drift and maintain a more stable Gmean trend. This further verifies that the proposed Gmean-based drift detection mechanism and adaptive forgetting factor effectively enhance the model’s adaptability to concept drift. Therefore, the revised manuscript strengthens the correspondence between the experimental results and the two main objectives of the proposed method, namely class imbalance handling capability and concept drift adaptation capability, thereby more clearly supporting the technical contributions of this study.

Attachment Submitted filename: Response to Reviewers.doc

# Editor report: Decision Letter 2

**DOI:** 10.1371/journal.pone.0353728.r005

**Authors:** Zeheng Wang

28 Jun 2026

Ensemble learning-based online sequential pre-interference extreme learning for concept drifting and class imbalanced data streams

PONE-D-25-66864R2

Dear Dr. Wen,

We’re pleased to inform you that your manuscript has been judged scientifically suitable for publication and will be formally accepted for publication once it meets all outstanding technical requirements.

Within one week, you’ll receive an e-mail detailing the required amendments. When these have been addressed, you’ll receive a formal acceptance letter and your manuscript will be scheduled for publication.

An invoice will be generated when your article is formally accepted. Please note, if your institution has a publishing partnership with PLOS and your article meets the relevant criteria, all or part of your publication costs will be covered. Please make sure your user information is up-to-date by logging into Editorial Manager at Editorial Manager® and clicking the ‘Update My Information' link at the top of the page. For questions related to billing, please contact billing support.

If your institution or institutions have a press office, please notify them about your upcoming paper to help maximize its impact. If they’ll be preparing press materials, please inform our press team as soon as possible -- no later than 48 hours after receiving the formal acceptance. Your manuscript will remain under strict press embargo until 2 pm Eastern Time on the date of publication. For more information, please contact onepress@plos.org.

Kind regards,

Zeheng Wang

Academic Editor

PLOS One

Additional Editor Comments (optional):

Reviewers' comments:

# Editor report: Acceptance letter

**DOI:** 10.1371/journal.pone.0353728.r006

**Authors:** Zeheng Wang

PONE-D-25-66864R2

PLOS One

Dear Dr. Wen,

I'm pleased to inform you that your manuscript has been deemed suitable for publication in PLOS One. Congratulations! Your manuscript is now being handed over to our production team.

At this stage, our production department will prepare your paper for publication. This includes ensuring the following:

* All references, tables, and figures are properly cited

* All relevant supporting information is included in the manuscript submission,

* There are no issues that prevent the paper from being properly typeset

You will receive further instructions from the production team, including instructions on how to review your proof when it is ready. Please keep in mind that we are working through a large volume of accepted articles, so please give us a few days to review your paper and let you know the next and final steps.

Lastly, if your institution or institutions have a press office, please let them know about your upcoming paper now to help maximize its impact. If they'll be preparing press materials, please inform our press team within the next 48 hours. Your manuscript will remain under strict press embargo until 2 pm Eastern Time on the date of publication. For more information, please contact onepress@plos.org.

You will receive an invoice from PLOS for your publication fee after your manuscript has reached the completed accept phase. If you receive an email requesting payment before acceptance or for any other service, this may be a phishing scheme. Learn how to identify phishing emails and protect your accounts at https://explore.plos.org/phishing.

If we can help with anything else, please email us at customercare@plos.org.

Thank you for submitting your work to PLOS ONE and supporting open access.

Kind regards,

PLOS ONE Editorial Office Staff

on behalf of

Dr. Zeheng Wang

Academic Editor

PLOS One
